# Robustification of GWAS to explore effective SNPs addressing the challenges of hidden population stratification and polygenic effects

**DOI:** 10.1038/s41598-021-90774-7

**Published:** 2021-06-22

**Authors:** Zobaer Akond, Md. Asif Ahsan, Munirul Alam, Md. Nurul Haque Mollah

**Affiliations:** 1 Bioinformatics Lab, Department of Statistics, University of Rajshahi, Rajshahi, 6205 Bangladesh; 2 Institute of Environmental Science, University of Rajshahi, Rajshahi, 6205 Bangladesh; 3 Molecular Ecology and Metagenomic Laboratory, Infectious Diseases Division, International Centre for Diarrheal Disease Research (Icddr,b), Rajshahi, Bangladesh; 4 Agricultural Statistics and ICT Division, Bangladesh Agricultural Research Institute (BARI), Gazipur, 1701 Bangladesh

**Keywords:** Computational biology and bioinformatics, Genetics, Plant sciences

## Abstract

Genome-wide association studies (GWAS) play a vital role in identifying important genes those is associated with the phenotypic variations of living organisms. There are several statistical methods for GWAS including the linear mixed model (LMM) which is popular for addressing the challenges of hidden population stratification and polygenic effects. However, most of these methods including LMM are sensitive to phenotypic outliers that may lead the misleading results. To overcome this problem, in this paper, we proposed a way to robustify the LMM approach for reducing the influence of outlying observations using the *β*-divergence method. The performance of the proposed method was investigated using both synthetic and real data analysis. Simulation results showed that the proposed method performs better than both linear regression model (LRM) and LMM approaches in terms of powers and false discovery rates in presence of phenotypic outliers. On the other hand, the proposed method performed almost similar to LMM approach but much better than LRM approach in absence of outliers. In the case of real data analysis, our proposed method identified 11 SNPs that are significantly associated with the rice flowering time. Among the identified candidate SNPs, some were involved in seed development and flowering time pathways, and some were connected with flower and other developmental processes. These identified candidate SNPs could assist rice breeding programs effectively. Thus, our findings highlighted the importance of robust GWAS in identifying candidate genes.

## Introduction

One of the major challenges in recent genetics research is to explore the biomarker genes that are linked to complex traits of interests in living organisms. Trait variations in living organisms are related to genetic variations in genes. These variations are observed largely at physiological, developmental, and morphological stages. Identification of important genetic basis such as causal genetic variants for such distinction in phenotypic traits is identifiable at single nucleotide polymorphism (SNP) levels. The techniques to explore the SNP contribution in phenotypic variation are termed as Genome-Wide Association Studies (GWAS). SNPs, however, are usually tested for relationship study through the whole genome with the characters of important trait of interest. The SNPs identified by GWAS can be used for the treatment and prevention of certain complex traits in living organisms. A very large set of SNPs along with a very large number of accessions are simultaneously studied using different GWAS methods to uncover the significant relationship between genomic latent factors and phenotypic variations of interest^1^.

Linear regression model (LRM) is a popular approach in GWAS. It is implemented through PLINK software for detecting important SNPs associated with quantitative trait^2^. The PLINK tool focused on parametric or nonparametric-based linear regressions which do not control population stratification. Population stratification is one of the main concerning issues when extensive genome-wide association analysis with numerous subjects is in consideration^3–5^. Some unidentified new population structures are probable to exist due to a large number of subjects that may perhaps liable for regular dissimilarities being selected in SNPs amongst cases and controls^4^. Due to the higher false discovery rate (FDR), it is imperative to correct the observed population stratification in GWAS^4,6^. There are, however, several statistical methodologies proposed earlier for GWAS to address the effects of population stratification. The most commonly used statistical methods to avoid the bias of population stratification (PS) or genetic relatedness are genomic control^7^, structured association^8^, and principal component analysis^9,10^. The genomic control (GC) method adjusts the statistical relationship by a common element concerning the whole set of SNPs for precise correction of PS^4^. If the consequence of population structure increases, the power of GC approach decreases^9,11–14^. The structured association (SA) analysis technique suggests locating the samples to separate subpopulation groups for collecting signs of a relationship in each group^8^. SA method is only useful for small datasets^4^. Principal Component Analysis (PCA) approach is also used to overcome the influence of population stratification in GWAS by using several topmost principal components (PCs) as covariates^4,9^. But none of the methods mentioned above can handle the influence of the polygenic effect. To overcome these issues, the linear mixed model (LMM) was proposed which is one of the most popular approaches in GWAS. It is widely using through several computer software such as TASSEL^13^, EMMA ^15^, EMMAX^16^, rrBLUP^17^, GAPIT^18^, and GAPIT Version 2^19^.

However, all the methods as early discussed are very much sensitive to phenotypic outliers. So, they can produce misleading results in presence of outlying observations. To overcome these issues, an attempt is made to robustify the LMM based GWAS by using a new type of outlier modification rule based on the minimum *β*-divergence method^20,21^. The performance of the proposed approach has been investigated using both simulated and real rice genome datasets related to flowering time.

## Results and discussion

We investigated the performance of the proposed method compare to two popular approaches (LMM and LRM) using both synthetic and real data analysis as discussed below:

### Results and discussion based on a complete simulation

To investigate the performance of SNPs detections with the synthetic datasets, we considered two original clean simulated datasets that were generated with heritabilities 0.2 and 0.3 respectively, as described in the materials and method section. We contaminated 1%, 2%, 3%, 4%, and 5% phenotypic observations by outliers to generate five contaminated datasets with each clean dataset to investigate the performance against the phenotypic outliers. Before going to the performance comparison, first, we would like to discuss the preprocessing steps for the proposed method as follows.

### Outlier detection and modification of phenotypic observations by the proposed method

To analyze these datasets by the proposed method, at first, we identified phenotypic outliers by using the *β*-weight function for each genotypic group and then replace the outlying phenotypic observations with the corresponding group mean computed by the minimum *β*-divergence method. To show how *β*-weight function detects outliers, we plotted *β*-weight corresponding to each phenotypic observation in the Supplementary Fig. S1 (a-b). Supplementary Fig. S1a consists of two panels, where the left panel plotted the original phenotypic observations and the right panel plotted their *β*-weights. Similarly, Supplementary Fig. S1b consists of two panels, where the left panel plotted the phenotypic observations including the 5% contaminated observations (red color), and the right panel plotted their *β*-weights. To select the outlying observations, we used the threshold value *τ*_*j*_ = *p*^*th*^ quantile value of the empirical distribution of $W_{\beta } (y_{li} |\hat{\theta }_{l,\beta } )$ as introduced in Eq. (7). We observed that the *β*-weight function correctly identified the outlying observations. Now we would like to discuss the consequence of outliers in the classical and proposed approaches by decomposing phenotypic variations as follows.

### The consequence of outliers on the partition of total phenotypic variations

To discuss the consequence outliers on the partition of total phenotypic variations for both classical and proposed approaches, we considered the original clean dataset including two contaminated datasets based on 2% and 5% outlying observations. Table 1 shows the consequence of outliers on the partition of total phenotypic variations for both classical and proposed approaches.

**Table 1:** Consequence phenotypic outliers on the partition of total variations with the classical and proposed approaches. The bold text indicates the partition of total variation for the clean dataset.

Sources of variations (SV)	Total phenotypic variation	Main genetic effect variation (Heritability)	Polygenic effect variation	Error variation	Rate of phenotypic outliers
	${\text{var}} (y)$	${\text{var}} \left( {\sum\limits_{k = 1}^{{{\text{m}}_{1} }} {{\text{a}}_{{\text{k}}} {\text{x}}_{{\text{k}}} } } \right)$	${\text{var}} \left( {\sum\limits_{{k = m_{1} + 1}}^{{{\text{m}}_{2} }} {{\text{b}}_{{\text{k}}} {\text{Z}}_{{\text{k}}} } } \right)$	${\text{var}} (\varepsilon)$	
In the case of Scenario-1	**Partition of total variation with the classical approach in presence of phenotypic outliers**				
	**100%**	**20%**	**40%**	**40%**	**0%** (clean data)
	100%	14.55%	26.34%	59.11%	2%
	100%	6.94%	12.56%	80.50%	5%
	**Partition of total variance by the proposed approach in presence of phenotypic outliers**				
	**100%**	**20%**	**40%**	**40%**	**0%** (clean data)
	100%	19.87%	39.27%	40.86%	2%
	100%	20.85%	41.37%	37.78%	5%
In the case of Scenario-2	**Partition of total variance by the classical approach in presence of phenotypic outliers**				
	**100%**	**30%**	**40%**	**30%**	**0%** (clean data)
	100%	12.37%	12.44%	77.19%	2%
	100%	7.72%	11.90%	80.38%	5%
	**Partition of total variance by the proposed approach in presence of phenotypic outliers**				
	**100%**	**30%**	**40%**	**30%**	**0%** (clean data)
	100%	29.64%	45.66%	24.70%	2%
	100%	28.99%	42.66%	28.34%	5%

We observed that variance proportions with respect to the genetic effect gradually decrease as increases the rate of outlying observations in the dataset by the classical approach, while the variance proportions with respect to the genetic effects are almost stable for each level of outlying rates in the dataset by the proposed approach. Therefore, the heritability (h^2*^) in presence of outliers becomes smaller than the heritability (h^2^) in absence of outliers by the classical approach, while the heritability ($\, \mathop h\nolimits_{\beta }^{2*} \,$) in presence of outliers is almost similar to the heritability (h^2^) in absence of outliers by the proposed approach.

### Performance comparison for SNPs detection

**Figure 1:**
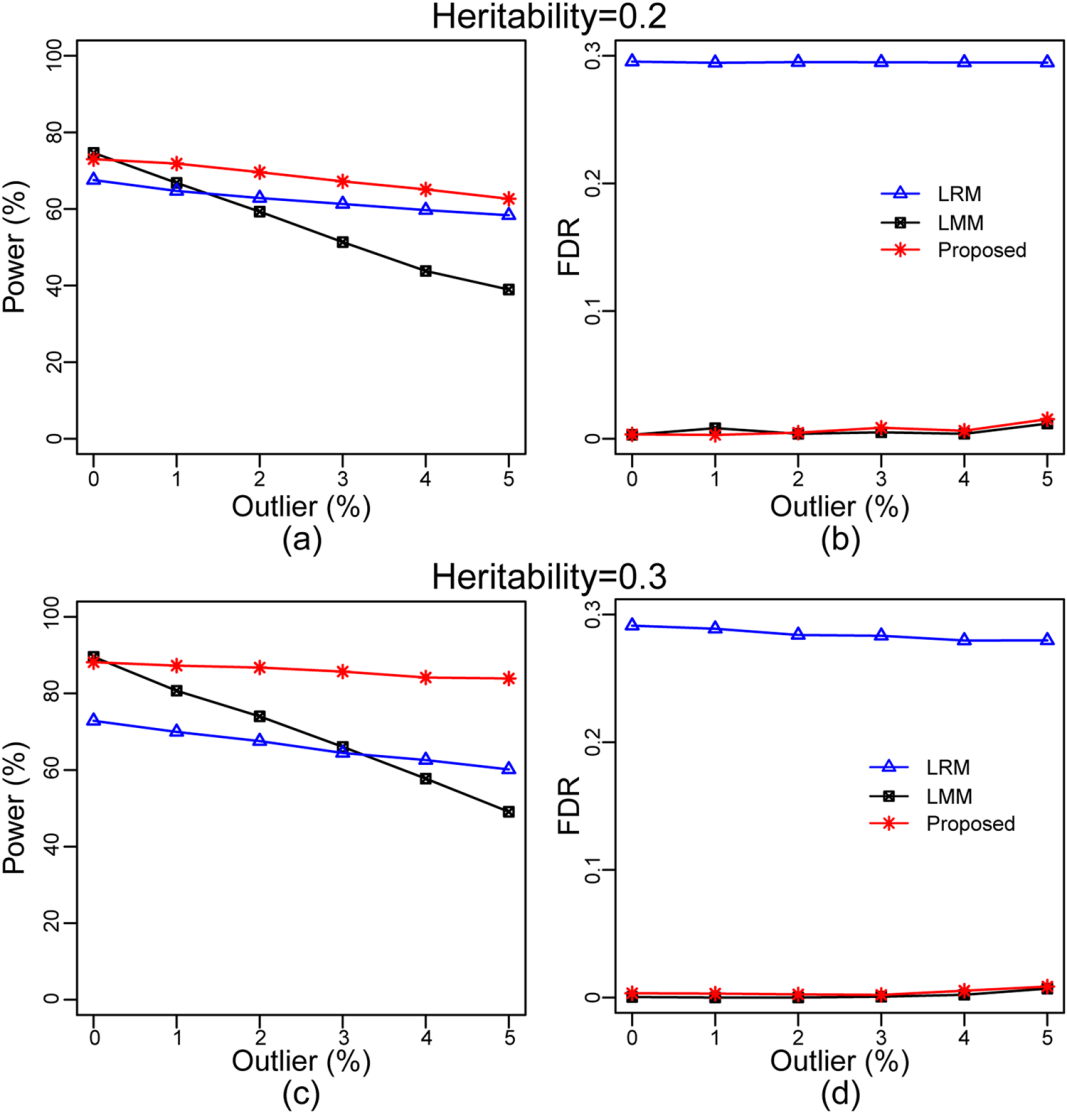
Results computed by LRM, LMM and the proposed methods based on complete simulation (**a**) plot of statistical power against the rate of phenotypic outliers at heritability h^2^ = 0.2 and at the cutoff *p*-value 10^–5^. (**b**) Plot of FDR against the rate of phenotypic outliers at h^2^ = 0.2 and at cutoff = 10^–5^. (**c**) plot of statistical power against the rate of phenotypic outliers at h^2^ = 0.3 and cutoff 10^–5^ (**d**) plot of FDR against the rate of phenotypic outliers at h^2^ = 0.3 and cutoff 10^–5^.

At first, we identified important SNPs by applying each of LRM, LMM, and the proposed methods on each of six datasets with each of two distinct genetic heritabilities 0.2 and 0.3 corresponding to the respective rates 0%, 1%, 2%, 3%, 4% and 5% of outliers. Then we computed statistical power and false discovery rate (FDR) for each of the methods to investigate the performance of the proposed method in a comparison of the classical LRM and LMM approaches. We computed average power and FDR based on 1000 replication of each dataset. Figure 1 showed the effect of outliers on statistical power and FDR with each of two distinct genetic heritabilities 0.2 and 0.3. It is observed from Figs. 1(a, c) that the power of the proposed method slowly decreased compare to the LMR and LMM approaches with the increasing rates of outliers. In absence of outliers, both the proposed and LMM approaches produced almost identical powers but much larger than the power of LRM. For example, in absence of outliers, the power of LRM, LMM and proposed methods were 67.55, 74.55 and 73.00 respectively for the dataset with heritability 0.2 (Fig. 1a), while 72.35, 89.95 and 88.65 respectively for the heritability 0.3 (Fig. 1c). However, with the increasing rates of outliers, the power of LMM approach decreased dramatically faster than the LRM approach. For example, in presence of 5% outliers with the phenotypic observations, the power of LRM, LMM and proposed methods were 58.35, 38.95 and 62.65 respectively for the dataset with heritability 0.2 (Fig. 1a), while 60.15, 49.10 and 83.90 respectively for the heritability 0.3 (Fig. 1c). Thus the, the proposed method produced much higher powers than both LRM and LMM for both scenarios of heritabilities in presence of outliers. The above two examples also indicated that the power of all three methods increase as the increasing of heritabilities in the datasets.

Figures 1(b,d) showed that FDRs of the proposed and LMM methods were almost same and close to zero in each rate of phenotypic outliers. However FDR for LRM was too high due to the influence of outliers along with the population stratification and polygenic effects^22^. Thus we may conclude that both LRM and LMM approaches are very much sensitive to phenotypic outliers compare to the proposed method. This results also supported by the decomposition of phenotypic variations described in Table 1, since power of any method decreases as the increasing of computational heritabilities.

### Performance comparison with some other robustification techniques

**Figure 2:**
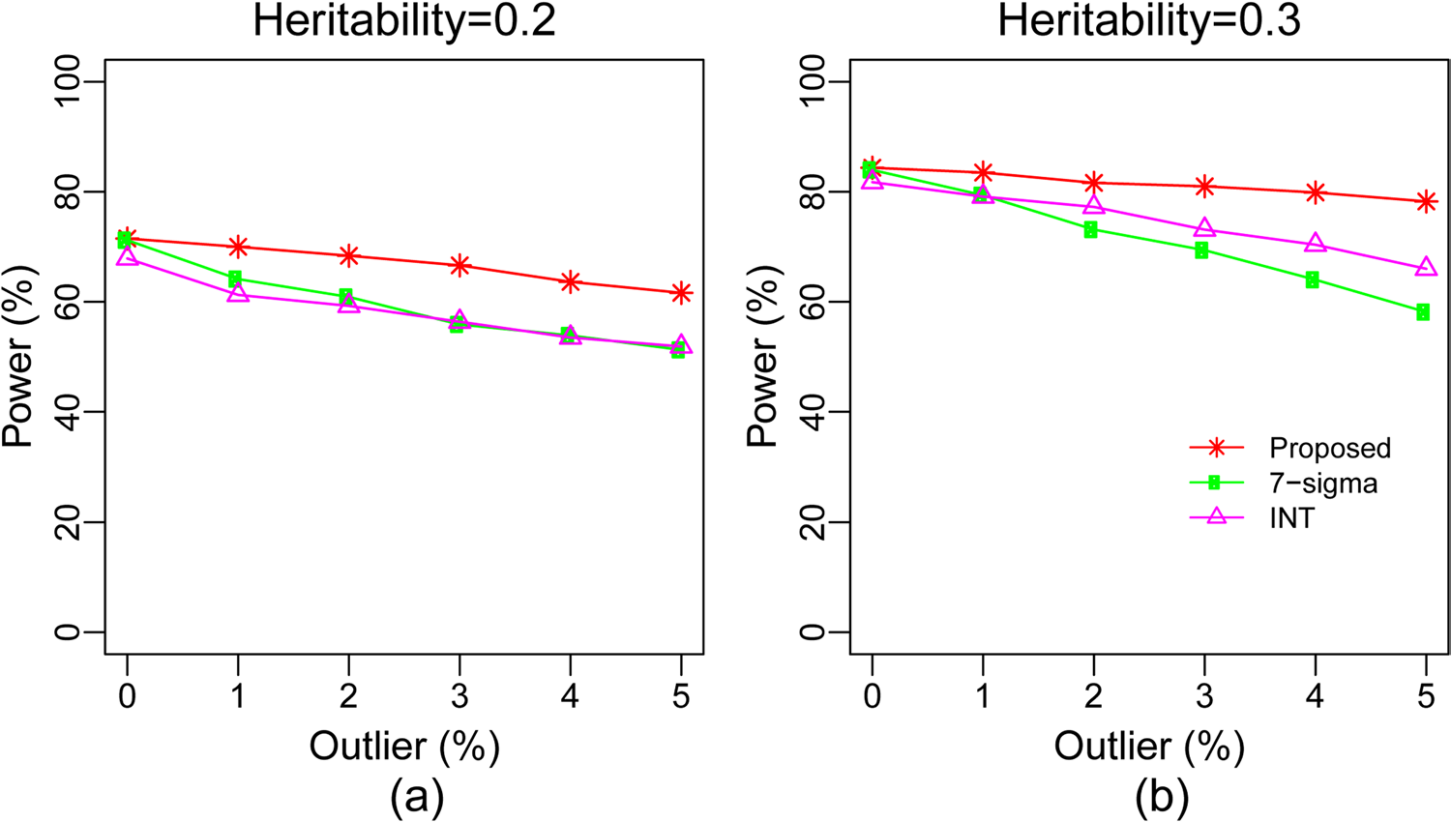
Plot of statistical power against the rate of phenotypic outliers based on the same dataset as used in Fig. 1 computed by three methods 7-sigmarule, inverse normal transformation (INT) and the proposed methods.

The proposed robustification technique was also compared with other two robustification techniques based on7-sigma rule ($\overline{y }\pm 7\sigma$) and inverse-normal transformation (INT)^23,24^ by the same datasets that were used in Fig. 1. We detected phenotypic outliers by the 7-sigma (7σ) rule and remove them before going to the analysis by LMM model. In the case of INT approach, we performed inverse-normal transformation on the phenotypic observations before going to the analysis by LMM model. In GWAS of quantitative traits/phenotypes, INT is commonly applied when the traits are distributed non-normally^24^. Figure 2 showed that the proposed method produces slightly higher power compare to 7σ and INT techniques for all cases of heritabilities and outliers. We also observed that the power of INT method is slightly higher compare to 7σ method in presence of outliers, but smaller in absence of outliers. There is one drawback with INT approach for the weak performance compare to the proposed method. The INT approach normalized the traits globally assuming the unimodal trait/phenotypic observations, while proposed method modified outliers corresponding to the genotypic groups assuming the multi-modal trait/phenotypic observations. Actually, phenotypic observations follow 3 modal distribution due to its 3 genotypes. There are two drawbacks with 7σ approach for the weak performance compare to both INT and the proposed methods. This method detected the outlying observations by assuming the unimodal distribution of phenotypic observations like INT approach and reduced the sample size by removing the outlying observation before going to the SNP detection by LMM model. The small sample size decreased the power significantly, which is satisfied by the previous study result^25^. Thus, the proposed robustification works well compare to 7σ and INT approaches.

### Performance comparison based on real SNP genotype and simulated phenotype data on rice flowering time

We also investigated the performance of the proposed method in a comparison of LRM and LMM based on partial simulation with the real SNP genotype and simulated phenotype data on rice flowering by considering the same condition like Fig. 1. The top four significant SNPs (id2005919, id2005983, ud2000772 and ud7002027) identified in real data analysis (See Table 2) were considered as main effect with the true effect size (a_k_). From the rest SNPs, we randomly selected 1996 SNPs as polygenic variants (effects). Finally the phenotypic observation were generated using Eq. (9).

**Table 2:** Identified 11 SNPs and the candidate genes of rice flowering time.

ID	*p*-value	Chr	Locus	Description
id2005644	2.44E-05	2	LOC_Os02g21070	PPR repeat domain-containing protein, putative, expressed
id2005743	4.62E-05	2	LOC_Os02g21880	coiled-coil domain-containing protein, putative, expressed
id2005919	2.63E-07	2	LOC_Os02g24134	Sec1 family transport protein, putative, expressed
ud2000772	1.82E-06	2	LOC_Os02g24770	retrotransposon protein, putative, Ty1-copia subclass, expressed
id2005983	4.88E-07	2	LOC_Os02g24780	retrotransposon protein, putative, unclassified, expressed
id2006587	8.89E-06	2	LOC_Os02g27750	transposon protein, putative, unclassified, expressed
wd6000761	3.44E-05	6	LOC_Os06g18000	protein kinase domain-containing protein, expressed
ud7002027	8.82E-06	7	LOC_Os07g45950	expressed protein
id8000022	2.11E-05	8	LOC_Os08g01070	retrotransposon protein, putative, unclassified, expressed
id8004076	4.92E-05	8	LOC_Os08g25040	expressed protein
id8004083	3.14E-05	8	LOC_Os08g25060	BSD domain-containing protein, putative, expressed

**Figure 3:**
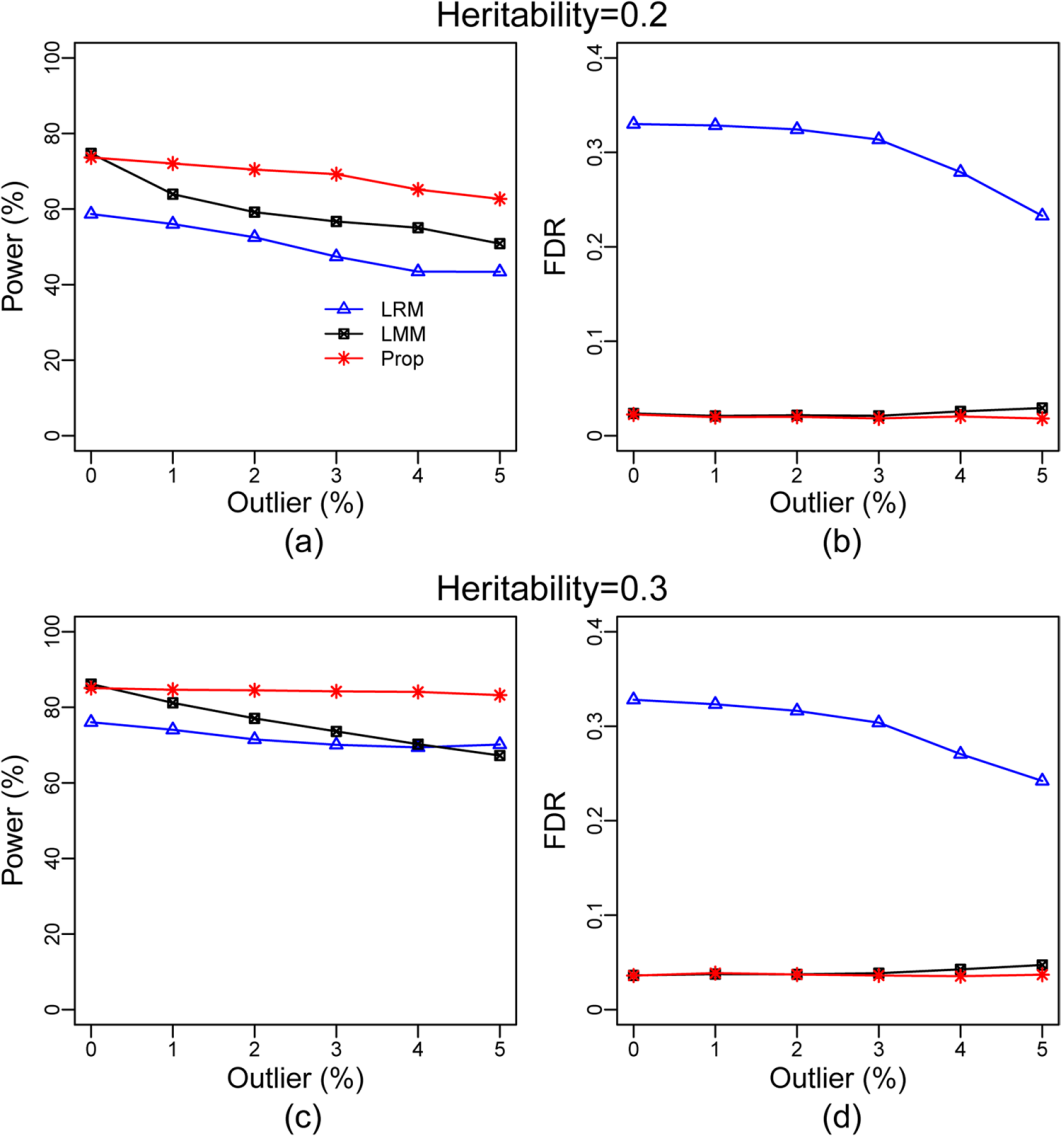
Results computed by LRM, LMM and the proposed methods based on partial simulation with the real dataset (**a**) plot of statistical power against the rate of phenotypic outliers at heritability h ^2^= 0.2 and at the cutoff *p*-value 10^–5^. (**b**) Plot of FDR against the rate of phenotypic outliers at h^2^ = 0.2 and at cutoff = 10^–5^. (**c**) plot of statistical power against the rate of phenotypic outliers at h^2^ = 0.3 and cutoff 10^–5^ (**d**) plot of FDR against the rate of phenotypic outliers at h^2^ = 0.3 and cutoff 10^–5^.

The Fig. 3 shows that the proposed method produces much larger power compare to both LRM and LMM which is supported by the results displayed in Fig. 1. However, with the increasing rate of phenotypic outliers, a slow decreasing rate of power was observed for the LMM method compared to LRM in Fig. 1. Again, with the increasing rate of phenotypic outliers, a decreasing trend of FDR for the LRM method was observed like Fig. 1d. This result could be due to the increase of outlier; the effect of population structure on the phenotype become weak. This result advocating that LMM method can control the confounding due to population stratification but not the outlier, whereas LRM method fail to control both the confounding and outlier. Thus, it may be concluded that the proposed method shows much better performance than the LRM and LMM in presence of phenotypic outliers; otherwise, it keeps the almost equal performance of LMM.

### Genome-wide association analysis of rice flowering time by the proposed method

**Figure 4:**
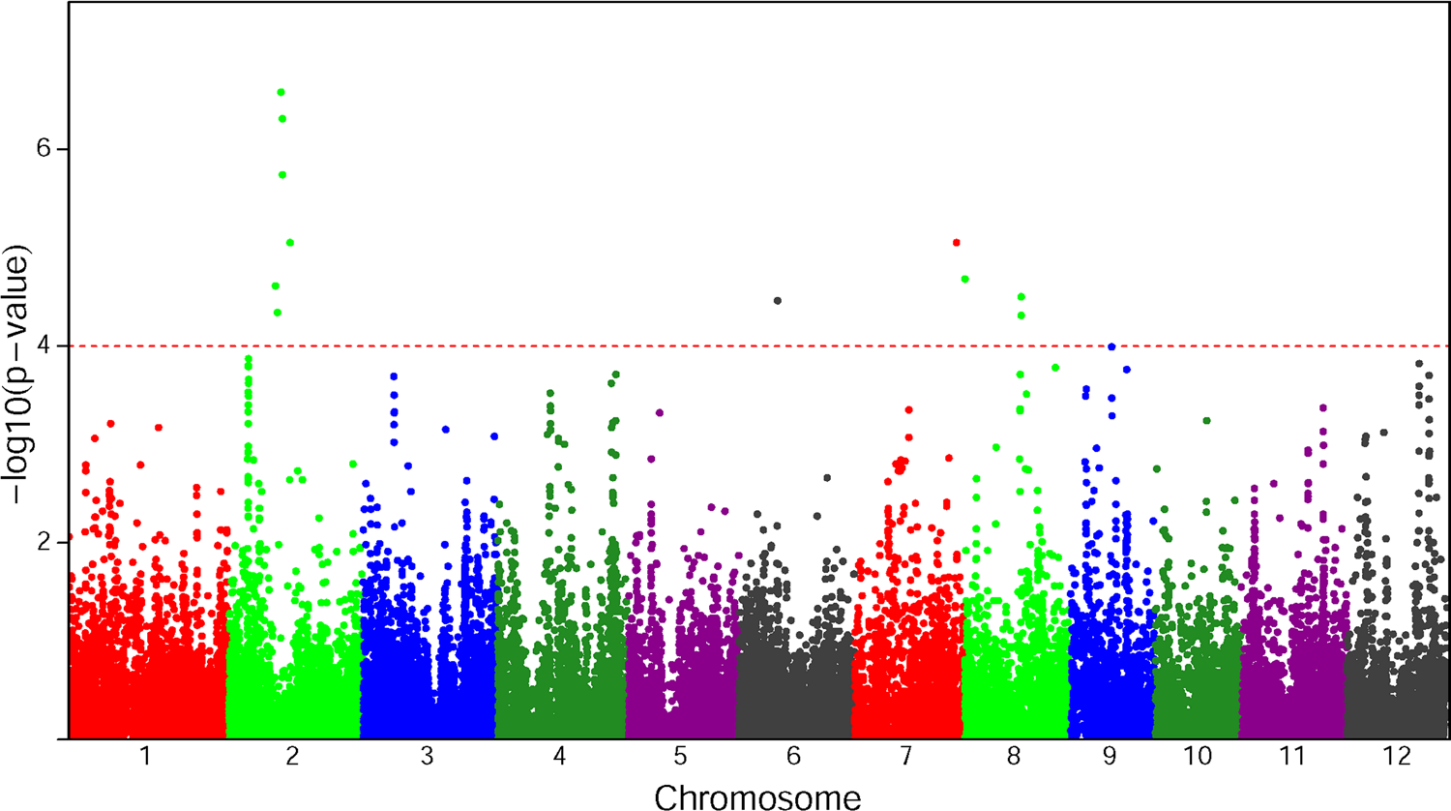
Manhattan plot using robust GWAS (rGWAS) on the trait of rice flowering time. The x-axis is the genomic position of the SNPs in the genome, and the y- axis is -log_10_ of the *p*-values. Each chromosome is colored differently. The grey horizontal line represents the minimal significant level at the cutoff.

We have analyzed the rice flowering time trait to identify the loci influencing this complex trait. Previous study by Zhao et al.^1^ on the same trait identified only 2 loci which motivated us to reanalyze the trait using our proposed robust GWAS (rGWAS) method. We have identified 11 significant SNPs using the proposed method (rGWAS) for rice flowering time with the threshold $p<9.99\times {10}^{-5}$ (Fig. 4). From the Manhattan plot it is observed that among the identified 11 SNPs, six SNPs lie in chromosome 2; one SNP marker belongs to each of the chromosome 6 and 7 and the rest three SNP markers belong to chromosome 8. However, these 11 SNPs did not overlap with the previously identified 2 SNPs. Zhow et al. used the genotype data with missing values as input in EMMA that omit the individuals with missing corresponding to the testing SNP. As a result the sample size was reduced that is responsible to decrease the detection power^25–27^. Moreover, the analyzed trait contained seven phenotypic outliers (Supplementary Fig. S2) that also responsible to reduce the statistical power (Fig. 1–2). A QQ-plot was also constructed using whole genome *p*-values of the proposed method. QQ-plot showed that the observed values correspond to the expected values are on or near the middle line between the x-axis and the y-axis (Supplementary Fig. S3) indicating that no genomic inflation has occurred in this analysis as well as population stratification is sufficiently controlled.

### Genomic information and characteristics of the candidate genes

To investigate the biological significance of the identified 11 SNPs, we validated these SNPs by using literature review and gold benchmark data. The corresponding chromosome and position of the identified SNPs were used to annotate and find the candidate genes comparing with the reference genome from the Rice Genome Annotation Project (RGAP) (http://rice.plantbiology.msu.edu/) database and collecting necessary genomic information given in Table 2 and Supplementary Table S1.

The SNP id2005644 was identified that located near the gene LOC_Os02g21070 which encodes pentatricopeptide repeats (PPR) domain-containing protein, and is assumed to take part in the biological molecule variation^28^. Plastid-localized pentatricopeptide repeat protein was reported in a study that is required for both pollen development and plant growth in rice^29^. A recent study in Arabidopsis showed that PPR containing protein affects flowering time^30^. Another variant id2005743 located in chromosome 2 and in the gene LOC_Os02g21880 was identified. The gene encodes coiled-coil domain-containing protein. This protein acts as the regulator of protein positioning in the cell during cell division by splitting and organizing signaling paths sequentially and spatially^31^. A coiled-coil domain containing protein in rice, PAIR1, was reported to express in the early stages of flower development^32^. Other four candidate genes (LOC_Os02g24770, LOC_Os02g24780 and LOC_Os02g27750, LOC_Os08g01070) were identified those encodes the retrotransposon and transposon transposable elements (TEs). Several studies have reported that these TEs can be induced by heat and cold stress in plants^33^. For example, Ty1-copia like retrotransposon ONSEN was found to be activated by heat stress in Arabidopsis^34^. Temperature and photoperiod are also found as two key regulatory factors associated with the flowering time in plants including rice^35^.

The detected SNPs were also mapped in the region of 100 kb of the genes those involved in the rice flowering time and seed development pathway^36^ and found five SNPs comprised of four genes involved in the two pathways (Supplementary Table S2). These results are suggesting the potential role of robust GWAS in detecting novel genes.

### Functional enrichment analysis of the candidate genes

**Figure 5:**
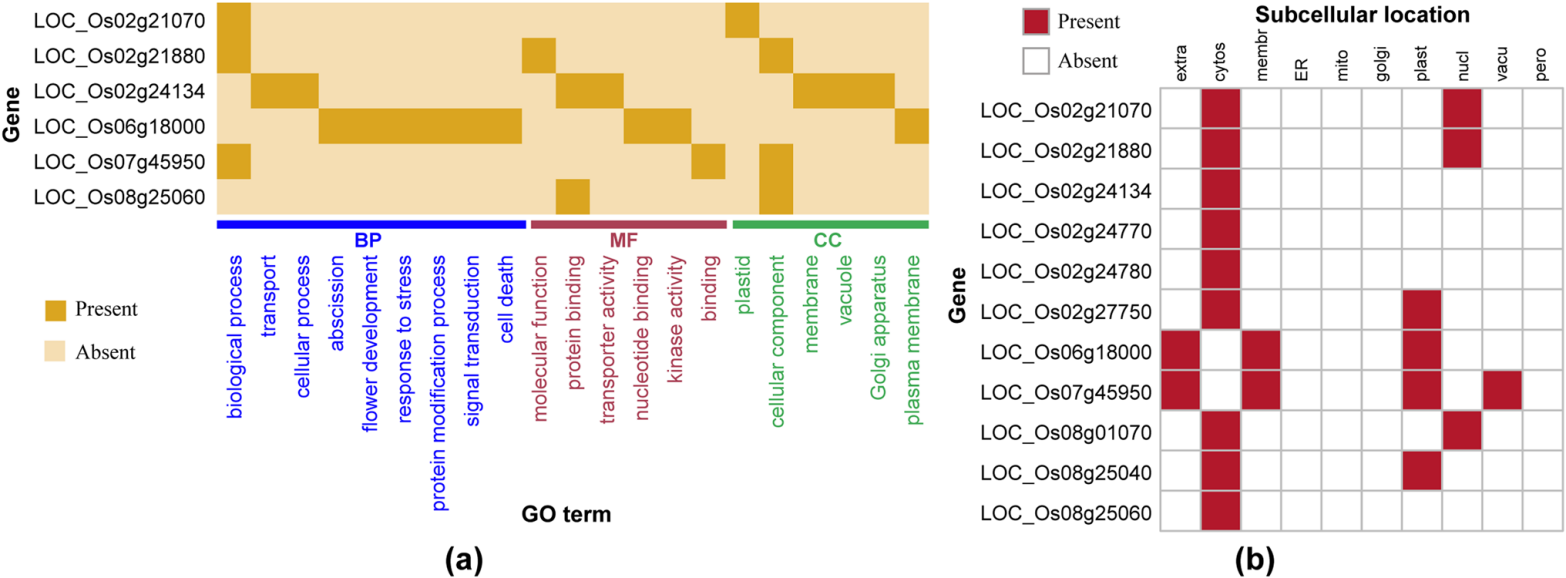
Expression map of GO and SCL for the 11 candidate genes. (**a**) Represents the expression map of the functional pathways viz., biological process (BP), molecular function (MF), and cellular component (CC) of the six candidate genes. (**b**) Represents the predicted subcellular location (SCL) of candidate genes in 10 molecular organs viz., cytosol (cytos), endoplasmic reticulum (ER), extracellular (extra), golgi apparatus (golgi), membrane (membr), mitochondria (mito), nuclear (nucl), peroxisome (pero), plastid (plast) and vacuole (vacu).

GO analysis is one of the major bioinformatics techniques for better understanding the underlying biological processes (BP) of the candidate genes along with their molecular functions (MF) and the cellular component (CC) of the genes^37,38^. Therefore, to more characterize the candidate genes, we have performed GO enrichment analysis and the results are shown in Fig. 5a and Supplementary Fig. S4a-c. The most important pathways or GO terms involving the candidate genes are highlighted with light yellow color in the rectangular boxes (Supplementary Fig. S4a-c). Flower development (GO:0,009,908), abscission (GO:0,009,838), signal transduction (GO:0,007,165), cell death (GO:0,008,219), cellular process (GO:0,009,987), response to stress (GO:0,006,950), and cellular protein modification (GO:0,006,464) were found as the most important pathways activated by the identified candidate genes in rice. Among the identified BPs, flower development (GO:0,009,908) is one of the crucial pathway that play the role for early and healthy grain development in rice^39,40^ and it is functionally linked to both the reproductive process (GO:0,022,414) and developmental process (GO:0,032,502) of rice plant (Fig. 5a and Supplementary Fig. S4a). Regulation of rice flowering time is delayed by several independent pathways and significantly influenced by prompt vegetative growth and reproductive process^41^. GO analysis showed that the shoot development (GO:0,048,367) and reproduction stages such as post-embryonic development (GO:0,009,791) are functionally related to rice flower development (Supplementary Fig. S4a).

Abscission, a part of a multicellular organism (GO:0,007,275), in GO enrichment analysis showed that it is directly related to the developmental process (GO:0,032,502) in rice. Earlier studies showed that seed shattering is controlled by the development of the abscission layer pathways in rice that allows offspring dispersal in the natural environment^42^. Signaling components via signal transduction (GO: 0,007,165) chain activate the different plant steroid hormones namely Brassinosteroids to regulate various growth and developmental programs, including cell differentiation and elongation, reproductive development, senescence, skotomorphogenesis (seedling development in the dark), and vascular differentiation^43–45^. The hormonal signal transduction pathways are also responsible to control several yield-related traits, including leaf angle, plant height, tiller number, and grain size in rice^43,44,46,47^. The predicted metabolic routes in this study may execute metabolism activities to convert food to energy to run cellular processes, to build blocks for proteins, lipids, nucleic acids, and some carbohydrates^48,49^. Some enzyme-catalyzed reactions may allow rice plants to grow and reproduce, maintain their structures, and respond to various stresses. The GO pathway, response to stress (GO:0,006,950) is predicted to play the role for controlling cellular activity in terms of movement, secretion, enzyme production, gene expression for the result of exogenous disturbance, temperature, humidity in rice plants.

### Subcellular location of the candidate genes

The cytosol is the place where the occurrence of the maximum different metabolisms in plants and most of the proteins in the cell are localized^50,51^. The predicted result of subcellular localization (SCL) of the candidate genes implied that nine genes out of eleven are localized in cytosol (Fig. 5b and Supplementary Data S1). Plastid is an important molecular organ found in plant cells mostly involve in photosynthesis and other gene expressions^52^. Photosynthesis is the key physiological parameter in rice that relates ultimately in many aspects to increase rice productivity^53^. Increase photosynthesis rate can utilize the solar radiation properly which leads to creating early flowering time because flowering signals are produced in leaves^41,54^. This gene expression in plastid likely to enhance the photosynthesis process, which regulates the leaf anatomy for earlier flowering in rice. Four candidate genes LOC_Os02g27750, LOC_Os06g18000, LOC_Os07g45950, and LOC_Os08g25040 were found in the plastid (plast). Out of the four genes the latter two genes were predicted to be located in extracellular (extra) and membrane (membr) of which LOC_Os07g45950 was predicted for vacuole (vacu) (Fig. 5b). It is also observed that three genes named LOC_Os02g21070, LOC_Os02g21880, and LOC_Os08g01070 are predicted to located in nuclear activity. However, no candidate genes were predicted to belong from the cellular locations viz., endoplasmic reticulum (ER), peroxisome (pero), and mitochondria (mito) (Fig. 5b).

### Expression profile of the candidate genes

**Figure 6:**
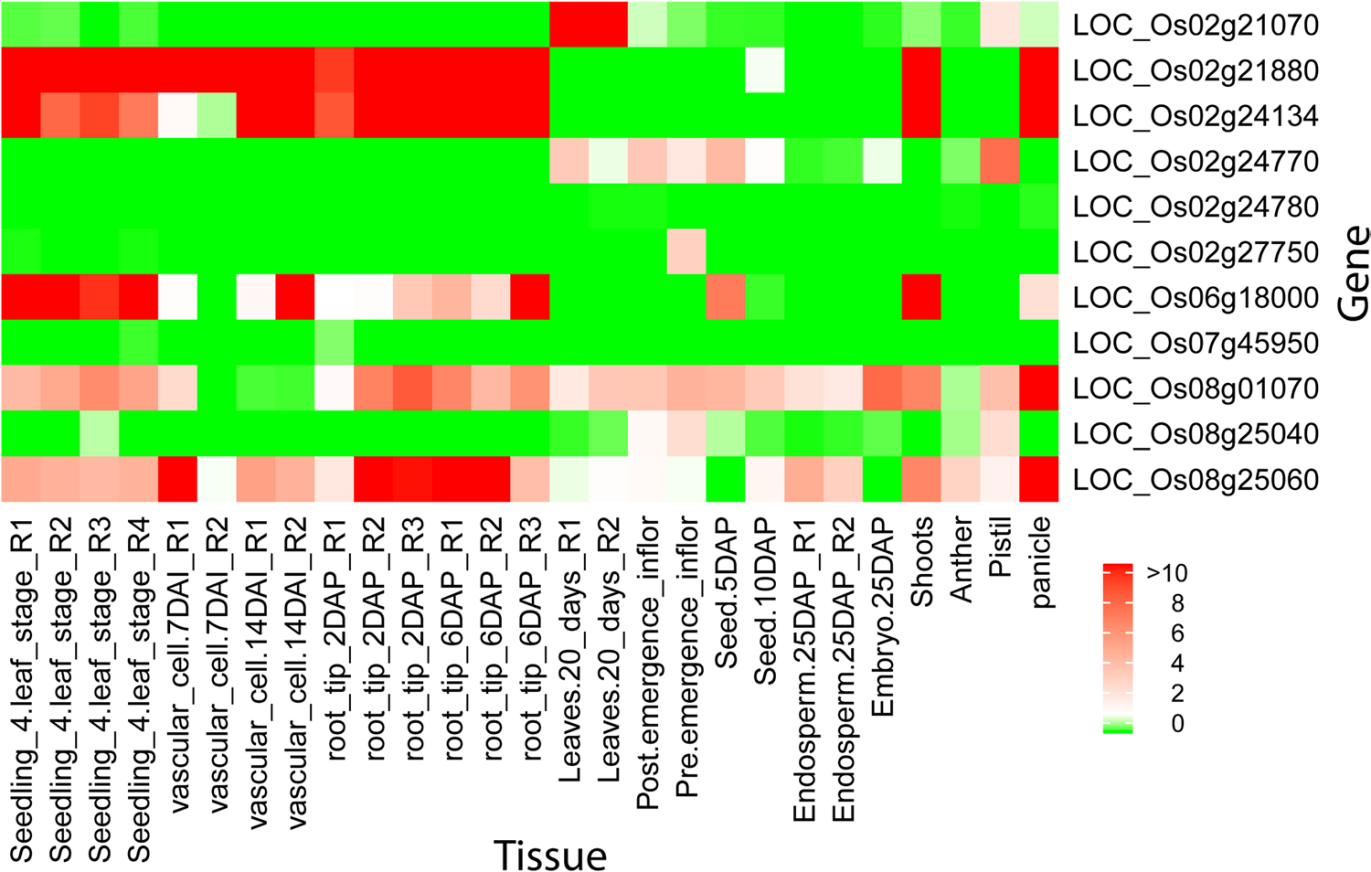
Heatmap showing the expression pattern of the identified 11 candidate genes. The heatmap represents the expression in various organs (seedling, vascular cell stage, root, leaves, post, and pre-emergence inflor, seed, endosperm, embryo, shoots, anther, pistil, and panicle) of rice. The color scale bar of the figure represents log2 transformed FPKM values.

The expression level of the candidate genes in different organs or tissues such as seedling, vascular cell, root, leaves, post-emergence, pre-emergence, seed, endosperm, embryo, shoots, anther, pistil, and panicle were extracted from the database Rice Genome Annotation Project (RGAP)^55^ (Supplementary Data S2). Heatmap presented in Fig. 6 exhibits the expression levels of the candidate genes through the organs or tissues. From the figure it was observed that the genes LOC_Os02g21880 and LOC_Os02g24134 showed high-level expression in seedling, root, shoot, and panicle in rice while these two genes exhibited only high-level expression in the vascular cell at 14DAP (Fig. 6). Moreover, seedling, vascular cell at 14DAP and shoots specific expressions were maximum for the gene LOC_Os06g18000. The genes LOC_Os02g21880, LOC_Os02g24134, LOC_Os08g01070 and LOC_Os08g25060 were found with high expression in panicle. The earlier study also suggested that leaves, shoot, and panicles have significant roles in regulating flowering time^41,56^. Our results obtained from real data analysis also consistent with earlier outcomes^1,22,31,57–59^.

## Conclusion

The GWAS is a powerful tool to explore the novel biomarker genes at the SNP level. The LMM approach has been considered as the leading statistical procedure to address the two main challenges of embedded population structures and genetic relatedness among individuals when GWAS is performed. It was however observed that this LMM approach is very much sensitive to phenotypic outlier that leads to misleading results. Therefore, in this article, we discussed a way to robustify the LMM procedure for controlling the outlying effect and reducing the puzzling effects of the population stratification and genetic relatedness in GWAS by using the minimum *β-*divergence method. This method introduced the *β*-weight function, which played a key role in the robustification procedure. For the convenience of the presentation, we call this method robust GWAS (rGWAS). Simulation results showed that the average power of correct identification of SNPs by LMM and the proposed method is almost the same and greater than 80%, which is much larger than the power of the linear regression model (LRM), in absence of outliers at heritabilities 0.2 and 0.3. The average power of LRM and the proposed method were slowly decreasing as gradually increasing the rate of phenotypic outliers with heritabilities 0.2 and 0.3, while the power of LMM was decreasing sharply and significantly. The false discovery rate (FDR) of LMM and proposed methods are almost the same and much smaller than the FDR of LRM in all cases of our simulation study. Thus, the proposed method outperformed the LRM and LMM in presence of outliers; otherwise, it shows the almost equal performance of LMM which is much better than LRM in absence of outliers. The demonstration of the proposed method with the real genome dataset against rice flowering time identified 11 important SNP makers. To investigate the biological significance of the identified 11 SNPs, we validated these SNPs by using literature review and gold benchmark data. We performed GSEA for the identified 11 SNP makers and SCL analysis to detect more valid SNPs out of 11 that have a significant association with the flowering time and other trait variations in rice. We also studied their expressions in various organs in rice to find the link with the flowering time. From GO analysis, it is observed that the gene LOC_Os06g18000 might play functional roles in flower development and response to stress in rice. Amongst the 11 genes, LOC_Os02g21880, LOC_Os06g18000, LOC_Os02g24134 exhibited larger expression in seedling, vascular cell, root, shoot, and panicle. Also, the gene LOC_Os08g25060 is predicted to provide maximum expression in vascular cell, root, and panicle. SCL results support that the cytosol contains the maximum number of genes. Plastid is an important molecular organ found in plant cells mostly involve in photosynthesis and other gene expressions. In our study, SCL analysis shows that the expression of the gene LOC_Os06g18000 in plastid may act as a flowering promoter. This gene expression in plastid likely to enhance the photosynthesis process which regulates the leaf anatomy for earlier flowering in rice. In GO analysis, it is also observed that this gene expression is associated with flowering in rice. Finally, it can be concluded that phenotypic outliers may significantly affect the analysis results in GWAS. Our proposed robust method outperforms the existing LRM and LMM methods in presence of outliers and the genomic information presented may however provide a basic platform for further biological investigations. To implement the proposed rGWAS method, the R-code and necessary instructions are available at the website.

## Materials and methods

### Robustification of LMM based GWAS by using the outlier modification rule (proposed)

The linear mixed model (LMM) approaches are extensively applied for genome-wide association studies (GWAS) for observable phenotypic variations in eukaryotic groups. If we consider that, there are *m* genotypes with *n* measurements of a phenotype. Efficient mixed-model association (EMMA)^15^ is such a model generally expressed by the following :

1 $$ {\varvec{y}} = {\varvec{Xa}} + {\varvec{Zb}} + {\varvec{\varepsilon}} $$

where ${\varvec{y}}\, = \,(y_{1} ,\,y_{2} ,...,y_{n} )^{\prime}$ is the *n* × *1* vector of phenotypic observations, and *X* = *(x*_*ij*_*)* is an *n* × *q* matrix of fixed effects including mean, SNPs, and other confounding variables.${\varvec{a}}$ is a *q* × *1* vector representing coefficients of the fixed effects. *Z* = *(z*_*ij*_*)* is an *n* × *m* incidence (design) matrix mapping each phenotype to one of the *m* genotypes. ***b*** is the vector of random polygenic effects which follows *N(0,σ*_*g*_^*2*^*K)*, where *σ*_*g*_^*2*^ is the polygenic variance component, and *K* = *(k*_*jt*_*)* is the *m* × *m* genomic relationship matrix. The genomic pairwise relationship coefficient between two individuals, *j* and *t*, is defined as follows

2 $$ k_{{jt}}  = \frac{1}{{T_{\varphi } }}\sum\limits_{{i = 1}}^{{T_{\varphi } }} {\frac{{(x_{{ij}}  - 2f_{i} )(x_{{it}}  - 2f_{i} )}}{{2f_{i} (1 - f_{i} )}}}  $$

where $T_{\varphi }$ is the total number of SNPs, *x*_*ij*_and *x*_*it*_ measure the numbers (0, 1, 2) of the minor allele(s) for the *i*^*th*^ SNP of the *j*^*th*^ and *t*^*th*^ individuals respectively, and *f*_*i*_ is the frequency of the minor allele. *ε* is the vector of random error which follows *N(0,σ*_*ε*_^*2*^*I),* where σ_ε_^2^ is the error variance component and where *I* is the *n* × *n* identity matrix. The overall phenotypic variance–covariance matrix can be represented as ${\mathbf{V}} = \sigma_{g}^{2} {\mathbf{ZKZ^{\prime}}} + \sigma_{\varepsilon }^{2} {\mathbf{I}}$.

where *I* is the *n* × *n* identity matrix. The variance components for polygenic effects and errors were estimated by restricted maximum likelihood (REML) using spectraldecomposition instead of the iterative expectation–maximization algorithm (EM algorithm)^15^. The full-likelihood function is maximized when ${\hat{a} = (X^{\prime}H}^{{{ - 1}}} {\mathbf{X}})^{ - 1} {\mathbf{X^{\prime}H}}^{ - 1} {\mathbf{y}}$ and the optimal variance component is $\hat{\sigma }_{F}^{2} = R/n$ for full-likelihood and $\hat{\sigma }_{R}^{2} = R/(n - q)$ for restricted likelihood, where $R = ({\varvec{y}} - {\varvec{Xa}})^{\prime}H^{ - 1} ({\varvec{y}} - {\varvec{Xa}})$ is a function of *δ* as well and $H = \sigma^{ - 1} V = {\varvec{Z}}K{\varvec{Z}} + \delta I$ is a function of *δ*, defined by $\delta = \sigma_{\varepsilon }^{2} /\sigma_{g}^{2} ,\,\,\,\sigma = \sigma_{g}$. When the maximum likelihood (ML) or restricted maximum likelihood (REML) variance component ${\hat{\mathbf{V}}} = \hat{\sigma }_{g}^{2} {\mathbf{ZKZ^{\prime}}} + \hat{\sigma }_{\varepsilon }^{2} {\mathbf{I}}$ is estimated, the classical *F*-statistic for testing the null hypothesis *Ma* = *0* for an arbitrary full-rank *p* × *q* matrix *M*^13,60^.

3 $$ F = \frac{{(M\hat{\user2{a}})^{\prime}(M({\varvec{X}}^{\prime}\hat{V}^{ - 1} {\varvec{X}})^{ - 1} M^{\prime})^{ - 1} (M\hat{\user2{a}})}}{p}\,\,\, $$

with *p* numerator degrees of freedom and *n-q* denominator degrees of freedom. The Satterthwaite degrees of freedom are calculated to avoid computationally intensive matrix operations. Both ML and REML estimators are very much sensitive if one or more phenotypic observations in ***y*** are outliers. Thus, LMM based *F*-statistic in Eq. (3) produces misleading results to test the null hypothesis in presence of outliers. In this study, we, therefore, consider the robustification of LMM based *F*-statistic in Eq. (3) by using the outlier identification and modification rule. There are some procedures for the identification of outliers in the literature; those are suitable when usual/clean data follows the unimodal distribution. However, in the current problem, phenotypic observations follow the multimodal distribution. So, conventional procedures cannot be used to identify the outlying observations properly. Therefore, in this paper, an attempt is made to propose a new outlier identification and modification rule by using the minimum *β*-divergence methods^20,61^ as follows:

(i) Select the top-ranking significant SNP associated with the phenotypic variations by using the minimum *β*-divergence based robust ANOVA^62^.

(ii) Divide the phenotypic data into *m* groups corresponding to the *m* genotypic labels of the selected most significant SNP. For example, let

$$ {\varvec{y}} = (y_{1} ,y_{2} ,...,y_{n} )^{\prime} = (y_{11} ,..,y_{{_{1} n_{1} }} ,...,\;y_{m1} ,..,y_{{mn_{m} }} )^{\prime} $$

be the partition of phenotypic observations corresponding to the selected SNP, where, *n* = *n*_*1*_ + *n*_*2*_ + *……..* + *n*_*m*_.

(iii) Detect the outlying observations from the *l*^*th*^ (*l* = *1,2,….,k*) group using the *β*-weight function defined by

4 $$ W_{\beta } (y_{li} |\hat{\theta }_{l} ) = exp\left\{ { - \frac{\beta }{{2\sigma_{li}^{2} }}(y_{li} - \hat{\mu }_{l} )^{2} } \right\} $$

where *i* = 1, 2, ... , *n*_*l*_

The minimum *β*-divergence estimators $\hat{\theta }_{l,\beta } = (\hat{\mu }_{l,\beta } ,\,\hat{\sigma }_{l,\beta }^{2} )$ of the parameters $\theta_{l,\beta } = (\mu_{l,\beta } , \,\sigma_{l,\beta }^{2} )$ are calculated iteratively by using the following formulas:

5 $$  \mu_{l,t + 1} = \frac{{\sum\limits_{i = 1}^{{n_{l} }} {W_{\beta } (y_{li} |\theta_{l,t} )y_{li} } }}{{\sum\limits_{i = 1}^{{n_{l} }} {W_{\beta } (y_{li} |\theta_{l,t} )} }} $$

and

6 $$  \sigma_{l,t + 1}^{2} = \frac{{\sum\limits_{i = 1}^{{n_{l} }} {W_{\beta } (y_{li} |\theta_{l,t} )(y_{li} - \mu_{l,t} )^{2} } }}{{(\beta + 1)^{ - 1} \sum\limits_{i = 1}^{{n_{l} }} {W_{\beta } (y_{li} |\theta_{l,t} )} }} $$

The notation *θ*_*t*+*1*_ denotes the update of $\theta_{t}$ in the *(t* + *1)th* iteration. The robustness of these estimators has been discussed in the background of influence function^20^ and their reliability^61^. It is noteworthy that the minimum *β*-divergence estimators $\hat{\theta }_{l,\beta } = (\hat{\mu }_{l,\beta } ,\hat{\sigma }_{l,\beta }^{2} )$ reduce to the classical maximum likelihood estimators (MLEs) $\hat{\theta }_{l} = (\hat{\mu }_{l} ,\hat{\sigma }_{l}^{2} )$ when *β* = 0.

It is considered that the MLEs of a Gaussian distribution are consistent and asymptotically efficient in absence of outlying objects^63^. Therefore, in this article, an effort has been provided to develop a robust linear mixed model (LMM) method in which the classical MLEs $\hat{\theta }_{l}$ are used in absence of outlying objects and minimum *β*-divergence estimators $\hat{\theta }_{l,\beta }$ stated in Eq. (5) and (6) are used in presence of outliers for estimation of *θ*_*l*_ in the mixed model. The minimum *β*-divergence method suggests two approaches for combining the robustness and efficiency of estimation in LMM. The tuning parameter *β* is selected through the cross-validation (CV) technique^20^. CV process produces *β* = 0 for the minimum *β*-divergence method estimators and is then equivalent to the classical estimators. When there are outlying subjects in the phenotypic traits, the technique generates *β* > 0 for the minimum *β*-divergence estimators. To overcome the challenges of outlying observations in GWAS, an alternative approach that is the *β*-weight function mentioned in (4) has been proposed with *β* = 0.2 for outlier detection. This weight function imposes smaller weights (≥ 0) to outlying observations and larger weights (≤ 1) to uncontaminated/usual objects.

An outlying phenotypic observation *y*_*li*_ in the *l*^*th*^ group is defined based on the *β*-weight function mentioned below:

7 $$ W_{\beta } (y_{li} |\hat{\theta }_{l,\beta } ) = \left\{ \begin{gathered}  >\tau_{l} ,{\text{if}}\;y_{li} \;{\text{is}}\;{\text{not}}\;{\text{an}}\;{\text{outlier}} \hfill \\ \le \tau_{l} ,\;{\text{if}}\;y_{li} \;{\text{is}}\;{\text{an}}\;{\text{outlier}} \hfill \\ \end{gathered} \right. $$

where the threshold value *τ*_*l*_ is the *p*^*th*^ quantile value of the empirical distribution of $W_{\beta } (y_{li} |\hat{\theta }_{l,\beta } )$.

(iv) Then replace the outlying phenotypic observations of *lth* group by its robust mean $\mu_{l,\beta }$(*l* = *1, 2,…,k*), where *m* is the number of genotype in the selected SNP.

(v) After that apply an efficient mixed-model association (EMMA) to the modified dataset discussed in the previous step.

### Simulated data generation

To investigate the performance of the proposed algorithm in a comparison of the conventional algorithms, we generated both the genotypic and phenotypic data as follows:

### Genotype simulation

To explore how the proposed method performs, a set of synthetic genotype and phenotype data were generated. A synthetic genotype dataset was simulated that reflects population structure. For this purpose, m* = 2000 SNPs were generated for *n* = 1000 individuals, and these individuals were taken from *k* = 3 distinct population by considering different minor allele frequencies (MAFs). To do this, first, a set of latent vectors {***v***_*1*_*, ****v***_*2*_*, …..****v***_m*_} was generated from a multivariate normal distribution with mean zero and variance–covariance matrix Cov*(v*_*j*_,*v*_*k*_) = *ρ*^*|j-k|*^^64,65^. In our simulation, we considered *ρ* = 0.5 to avoid the linkage disequilibrium (LD) between the SNPs. Finally, two cutoff values *s*_*1*_ and *s*_*2*_ were used to convert the design matrix ***V*** = [***v***_*1*_*, ****v***_*2*_*, …, ****v***_m*_ ] = [*v*_*ij*_] of latent vectors to the genotypic score matrix $x_{ij} (i = 1,2,...,n,j = 1,2,...,m_{1} )\,{\text{and }}\,z_{ij} (i =  1,2,...,n;j = 1,2,...,m_{2} )$ as follows:

$$ x_{ij} ,z_{ij} = \left\{ \begin{gathered} 0,\;\;\;\;\;v_{ij} < s_{1} \hfill \\ \,\,1,\;\;\;\;\;s_{1} \le v_{ij} \le s_{2} \hfill \\ \,\,2,\;\;\;\;\;v_{ij} > s_{2} \hfill \\ \end{gathered} \right. $$

where *s*_*1*_ and *s*_*2*_ determine the minor allele frequency.

### Phenotype simulation

Phenotypic datasets were produced by considering several factors including genetic variation, error variation, and population stratification. To generate phenotype data, two distinct situations were considered in terms of two heritability rates 0.2 and 0.3. In every situation, *m*_*1*_ = 4 SNPs were considered as causal variants and the remaining *m*_*2*_ = m*-m_*1*_ = *1996* SNPs were allocated as polygenic variants (effects). The quantitative trait/phenotypic values were simulated using Eq. (1) which can be re-written as

8 $$ \mathop y\nolimits_{j} = \mu + \sum\limits_{k = 1}^{{m_{1} }} {a_{k} x_{kj} } + \sum\limits_{{k = m_{1} + 1}}^{{m_{2} }} {b_{k} z_{kj} } + \mathop \varepsilon \nolimits_{j} \,\,\, $$

The total phenotypic variation was decomposed by assuming all three sources of variations in Eq. (8) are independent each other as

9 $$ {\text{var}} (y) = {\text{var}} \left( {\sum\limits_{k = 1}^{{m_{1} }} {a_{k} x_{k} } } \right) + {\text{var}} \left( {\sum\limits_{{k = m_{1} + 1}}^{{m_{2} }} {b_{k} z_{k} } } \right) + {\text{var}} (\varepsilon)$$

Then the contribution of main genetic effect in the total phenotypic variation (known as heritability) was defined by the ratio of main genetic variance over the phenotypic variance and it is written as

10 $$ {h}^{2} = \frac{{{\text{var}} \left( {\sum\limits_{k = 1}^{{m_{1} }} {a_{k} x_{k} } } \right)}}{{{\text{var}} (y)}}\,\,\,\,\,\, $$

The genomic outcomes of the SNPs were simulated from a normal distribution such that it satisfies a certain proportion of genetic variance for different genetic effects as given in Table 3.

**Table 3:** Distribution of different genetic variation in the phenotype.

Scenario	Genotype data	Main effect variation	Polygenic effect variation	Total genetic variation	Error variation
		${\text{var}} \left( {\sum\limits_{k = 1}^{{m_{1} }} {\rm a_{\rm k} \rm x_{\rm ki} } } \right)$	${\text{var}} \left( {\sum\limits_{k = m_{1}+1}^{{{\text{m}}_{2}}} {\rm b_{\rm k} \rm z_{\rm k}}} \right)$		${\text{var}} (\mathop \varepsilon \nolimits)$
Scenario I	Simulated	20%	40%	60%	40%
Scenario II		30%	40%	70%	30%
Scenario I	Real	20%	40%	60%	40%
Scenario II		30%	40%	70%	30%

To check the performance of the proposed method in a comparison of LRM and LMM approaches in presence of different rate of outliers, we contaminated 1%, 2%, 3%, 4%, and 5% of phenotypic data randomly by using theoutlying observations to generate five different contaminated datasets. We replicated these five contaminated datasets including original clean dataset 1000 times. Tthe outlying phenotypic observations was generated $\left( {\mathop y\nolimits_{j}^{*} } \right)$ satisfying ${\text{2}} \times {\mathbf{max}}({\mathbf{y}}){\mkern 1mu}  < {\text{y}}_{j}^{*}  < {\text{5}} \times \max ({\mathbf{y}})$.

### Consequence of phenotypic outliers on the partition of total variations

Let y* be the modified phenotypic response variable which is generated by replacing 5% observation of y by the outlying observations. Then var(y*) > var(y) obviously, which implies

11 $$ {\text{var}} \left( {\mathop y\nolimits^{*} } \right) = {\text{var}} \left( y \right) + c,\,\,\, $$

where c > 0. Now we can write the following equation similar to the Eq. (8) as

12 $$ \mathop y\nolimits_{j}^{*} = \mu + \sum\limits_{k = 1}^{{m_{1} }} {a_{k} x_{kj} } + \sum\limits_{k = 1}^{{m_{2} }} {b_{k} z_{kj} } + \mathop \varepsilon \nolimits_{j}^{*} \,\,\,\, $$

by assuming the same genetic effects but changed to the error variations. Then

13 $$ {\text{var}} \left( {\mathop y\nolimits^{*} } \right) = {\text{var}} \left( {\sum\limits_{k = 1}^{{m_{1} }} {a_{k} x_{k} } } \right) + {\text{var}} \left( {\sum\limits_{{k = m_{1} + 1}}^{{m_{2} }} {b_{k} z_{k} } } \right) + {\text{var}} \left( {\mathop \varepsilon \nolimits^{*} } \right)\,\,\,\, $$

Combining Eq. (9), (11) and (13), we get

$$ {\text{var}} \left( {\mathop \varepsilon \nolimits^{*} } \right) = {\text{var}} (\varepsilon ) + c > {\text{var}} (\varepsilon ) $$

Thus, error variance in presence of outliers must be larger than the error variance in absence of outliers. Conversely, the heritability (h^2*^) in presence of outliers must be smaller than the heritability (h^2^) in absence of outliers as follows.

$$ h^2 = \frac{{{\text{var}} \left( {\sum\limits_{k = 1}^{{m_{1} }} {a_{k} x_{k} } } \right)}}{{{\text{var}} (\mathop y\nolimits_{{}} )}} > \frac{{{\text{var}} \left( {\sum\limits_{k = 1}^{{m_{1} }} {a_{k} x_{k} } } \right)}}{{{\text{var}} (\mathop y\nolimits_{{}}^{*} )}} = \mathop h\nolimits^{2*} $$

Therefore, both LMM and LRM approach losses the SNPs identification power in presence of phenotypic outliers. However, in the case of the proposed method, the error variance in presence of outliers must be almost same to the error variance in absence of outliers. Similarly, the heritability $\, \mathop h\nolimits_{\beta }^{*} \,$ in presence of outliers must be almost same to the heritability (h^2^) in absence of outliers, since

$$ {\text{var}} \left( {\mathop y\nolimits_{\beta }^{*} } \right) \approx {\text{var}} \left( {\mathop y\nolimits_{{}}^{{}} } \right), $$

where ${\text{var}} \left( {\mathop y\nolimits_{\beta }^{*} } \right)$ is the variance of total phenotypic variations after the preprocessing of phenotypic observations by the proposed method. Therefore, the power of the proposed method must be larger than the power of both LMM and LRM methods in presence of phenotypic outliers.

## Performance measures

To investigate the performance, the statistical power and FDR of 3 methods were calculated by using the formula, Power = (*P*_*T*_*/P*_*C*_) × 100 and FDR = [*P*_*F*_/(*P*_*T*_ + *P*_*F*_)] × 100, respectively, where *P*_*T*_ measures the truly detected SNPs and *P*_*C*_ measures total causal variants and *P*_*F*_ is the number of falsely detected SNPs. For each situation, 1000 replications were performed to account for the average value of the power and FDR for comparison.

### Real genotype and phenotype data on rice flowering time

We applied the proposed rGWAS method to explore the potential SNPs influencing rice flowering time. The genotypic and phenotypic data used to carry out the analysis for investigation were collected from the rice diversity database (www.ricediversity.org). The data set contain 413 accessions along with 36,901 SNPs of *Oryza Sativa*^1^. All selected SNPs were taken into consideration in the analysis with call rate > 70% and minor allele frequency (MAF) > 0.05^1^. Missing genotypes were imputed with weighted k-Nearest-Neighbors method^66^ based on the five weighted nearest varieties. To compute the kinship matrix (using Eq. (2)), LD-pruned set of variants was used with an R^2^ greater than 0.9 in a 200 variant sliding window of size 1000. The individual with missing observation in the phenotypic dataset was not considered in this study. Experimental data on flowering time were obtained as the number of days until the inflorescence was 50% emerged from the flag leaf calculated from the day of planting. The phenotypic data used in this analysis for the flowering time were recorded at Faridpur district in Bangladesh.

### SNP annotation and candidate gene identification

11 SNPs were identified through whole genome association analysis by using rGWAS. Rice Genome Annotation Project (RGAP) Release 7 (http://rice.plantbiology.msu.edu/) database was used to annotate the identified SNPs. Among the identified genes, six were annotated with protein-coding genes and the rest five were non-coding (Supplementary Table S1). For the non-coding SNPs, the nearest genes were considered as candidate gene and used for further functional characterization. We also aligned the significant SNP tags against the genes those involved in rice flowering time and seed development pathway^36^. Region within 100 kb were searched for the pathway genes (Supplementary Table S2). The QQ-plot were generated using qqmath function in the R package *lattice*^67^.

### Gene-set enrichment analysis

To characterized the candidate genes/SNPs that may have a significant association with the phenotypic variations, we performed gene ontology (GO) enrichment analysis of the candidate genes in terms of biological process (BP), molecular function (MF), and cellular component (CC) were performed using the on-line tool QuickGO (https://www.ebi.ac.uk/QuickGO). A gene set was considered as significantly enriched for GO terms if $p<0.05$. The heatmap of the GO corresponding to the candidate genes was plotted using R package *ComplexHeatmap*^68^.

### Prediction of the subcellular location

An online-based tool called Plant Subcellular Localization Integrative (PSI) predictor^69^ was used for predicting the subcellular locations of the candidate genes in the plant cell. The predicted subcellular location corresponding to the candidate genes were visualize by using the R package *corrplot*^70^.

### Tissue-specific expression

We explored the expression profile of the candidate genes in different tissues. Tissue-specific expression of the genes were obtained from the database Rice Genome Annotation Project (RGAP)^55^. The heatmap of the expression level of the genes was created via R package *ComplexHeatmap*^68^.

### Data availability

To implement the proposed method, the necessary codes in R can be downloaded from the repository:http://www.ru.ac.bd/biorgru/software/r-code-robustgwas-zip/

## Supplementary Information

Supplementary Information 1.

Supplementary Information 2.

Supplementary Information 3.

## References

[1] Zhao K (2011). Genome-wide association mapping reveals a rich genetic architecture of complex traits in Oryza sativa. Nat. Commun..

[2] Purcell S (2007). PLINK: A tool set for whole-genome association and population-based linkage analyses. Am. J. Hum. Genet..

[3] Li Q, Yu K (2008). Improved correction for population stratification in genome-wide association studies by identifying hidden population structures. Genet. Epidemiol..

[4] Liu L, Zhang D, Liu H, Arendt C (2013). Robust methods for population stratification in genome wide association studies. BMC Bioinformatics.

[5] Xu H, Sarkar B, George V (2009). A new measure of population structure using multiple single nucleotide polymorphisms and its relationship with FST. BMC. Res. Notes.

[6] Campbell CD (2005). Demonstrating stratification in a European American population. Nat. Genet..

[7] Devlin B, Roeder K (1999). Genomic control for association studies. Biometrics.

[8] Pritchard JK, Stephens M, Rosenberg NA, Donnelly P (2002). Association mapping in structured populations. Am. J. Hum. Genet..

[9] Price AL (2006). Principal components analysis corrects for stratification in genome-wide association studies. Nat. Genet..

[10] Patterson N, Price AL, Reich D (2006). Population structure and eigenanalysis. PLoS Genet..

[11] Aranzana MJ (2005). Genome-wide association mapping in Arabidopsis identifies previously known flowering time and pathogen resistance genes. PLoS Genet..

[12] Devlin B, Roeder K, Wasserman L (2001). Genomic control, a new approach to genetic-based association studies. Theor. Popul. Biol..

[13] Yu J (2006). A unified mixed-model method for association mapping that accounts for multiple levels of relatedness. Nat. Genet..

[14] Zhao K (2007). An Arabidopsis example of association mapping in structured samples. PLoS Genet..

[15] Hyun MK (2008). Efficient control of population structure in model organism association mapping. Genetics.

[16] Kang HM (2010). Variance component model to account for sample structure in genome-wide association studies. Nat. Genet..

[17] Endelman JB (2011). Ridge regression and other kernels for genomic selection with R package rrBLUP. Plant Genome J..

[18] Lipka AE (2012). GAPIT: Genome association and prediction integrated tool. Bioinformatics.

[19] Tang Y (2016). GAPIT Version 2: an enhanced integrated tool for genomic association and prediction. Plant Genome.

[20] Mollah MNH, Eguchi S, Minami M (2007). Robust prewhitening for ICA by minimizing β-divergence and its application to FastICA. Neural Process. Lett..

[21] Mollah MNH, Eguchi S (2010). Robust QTL analysis by minimum &beta;-divergence method. Int. J. Data Min. Bioinform..

[22] Ahsan A (2018). Identification epistasis loci underlying rice flowering time by controlling population stratification and polygenic effect. DNA Res..

[23] Beasley TM, Erickson S, Allison DB (2009). Rank-based inverse normal transformations are increasingly used, but are they merited?. Behav. Genet..

[24] McCaw ZR, Lane JM, Saxena R, Redline S, Lin X (2020). Operating characteristics of the rank-based inverse normal transformation for quantitative trait analysis in genome-wide association studies. Biometrics.

[25] Jiang W, Yu W (2016). Power estimation and sample size determination for replication studies of genome-wide association studies. BMC Genom..

[26] Wang M, Xu S (2019). Statistical power in genome-wide association studies and quantitative trait locus mapping. Heredity.

[27] Hong EP, Park JW (2012). Sample size and statistical power calculation in genetic association studies. Genom. Inf..

[28] Sharma M, Pandey GK (2016). Expansion and function of repeat domain proteins during stress and development in plants. Front. Plant Sci..

[29] Liu YJ (2017). A plastid-localized pentatricopeptide repeat protein is required for both pollen development and plant growth in rice. Sci. Rep..

[30] Emami H, Kempken F (2019). PRECOCIOUS1 (POCO1), a mitochondrial pentatricopeptide repeat protein affects flowering time in Arabidopsis thaliana. Plant J..

[31] Rose A (2004). Genome-wide identification of arabidopsis coiled-coil proteins and establishment of the ARABI-COIL database. Plant Physiol..

[32] Nonomura KI (2004). The novel gene homologous pairing aberration in rice Meiosis1 of rice encodes a putative coiled-coil protein required for homologous chromosome pairing in meiosis. Plant Cell.

[33] Ito H (2016). A stress-activated transposon in arabidopsis induces transgenerational abscisic acid insensitivity. Sci. Rep..

[34] Ito H (2011). An siRNA pathway prevents transgenerational retrotransposition in plants subjected to stress. Nature.

[35] Craufurd PQ, Wheeler TR (2009). Climate change and the flowering time of annual crops. J. Exp. Bot..

[36] Hanumappa M (2013). WikiPathways for plants: a community pathway curation portal and a case study in rice and arabidopsis seed development networks. Rice.

[37] Harris MA (2008). The gene ontology project in 2008. Nucleic Acids Res..

[38] Harris MA (2004). The gene oncology (GO) database and informatics resource. Nucleic Acids Res..

[39] Huang X (2012). Genome-wide association study of flowering time and grain yield traits in a worldwide collection of rice germplasm. Nat. Genet..

[40] Cho LH, Yoon J, An G (2017). The control of flowering time by environmental factors. Plant J..

[41] Lee YS, An G (2015). Regulation of flowering time in rice. J. Plant Biol..

[42] Zhou Y (2012). Genetic control of seed shattering in rice by the APETALA2 transcription factor Shattering Abortion1. Plant Cell.

[43] Zhang C, Bai M, Chong K (2014). Brassinosteroid-mediated regulation of agronomic traits in rice. Plant Cell Rep..

[44] Mori M (2002). Isolation and characterization of a rice dwarf mutant with a defect in brassinosteroid biosynthesis. Plant Physiol..

[45] Clouse SD, Langford M, McMorris TC (1996). A brassinosteroid-lnsensitive mutant in arabidopsis thaliana exhibits multiple defects in growth and development. Plant Physiol..

[46] Divi UK, Krishna P (2009). Brassinosteroid: a biotechnological target for enhancing crop yield and stress tolerance. New Biotechnol..

[47] Yamamuro C (2000). Loss of function of a rice brassinosteroid insensitive1 homolog prevents internode elongation and bending of the lamina joint. Plant Cell.

[48] Smith E, Morowitz HJ (2004). Universality in intermediary metabolism. Proc. Natl. Acad. Sci. U.S.A..

[49] Smith RL, Soeters MR, Wüst RCI, Houtkooper RH (2018). Metabolic flexibility as an adaptation to energy resources and requirements in health and disease. Endocr. Rev..

[50] Kholodenko BN (2003). Four-dimensional organization of protein kinase signaling cascades: the roles of diffusion, endocytosis and molecular motors. J. Exp. Biol..

[51] Ohlrogge JB, Kuhn DN, Stumpf PK (1979). Subcellular localization of acyl carrier protein in leaf protoplasts of Spinacia oleracea. Proc. Natl. Acad. Sci. U.S.A..

[52] Jansen RK (2005). Methods for obtaining and analyzing whole chloroplast genome sequences. Methods Enzymol..

[53] Hidayati, N., Triadiati & Anas, I. Photosynthesis and transpiration rates of rice cultivated under the system of rice intensification and the effects on growth and yield. *HAYATI J. Biosci.* (2016). doi:10.1016/j.hjb.2016.06.002

[54] Karki, S., Rizal, G. & Quick, W. P. Improvement of photosynthesis in rice (Oryza sativa L.) by inserting the C4 pathway. *Rice* (2013). doi:10.1186/1939-8433-6-28 10.1186/1939-8433-6-28 PMC4883725 24280149

[55] Kawahara Y (2013). Improvement of the oryza sativa nipponbare reference genome using next generation sequence and optical map data. Rice.

[56] Weng X (2014). Grain number, plant height, and heading date7 is a central regulator of growth, development, and stress response. Plant Physiol..

[57] Shivani *et al.* Genome-wide analysis of transcription factors during somatic embryogenesis in banana (Musa spp.) cv. Grand Naine. *PLoS ONE* (2017). doi:10.1371/journal.pone.0182242 10.1371/journal.pone.0182242 PMC5552287 28797040

[58] Pasam RK (2012). Genome-wide association studies for agronomical traits in a world wide spring barley collection. BMC Plant Biol..

[59] Assaad, F. F., Huet, Y., Mayer, U. & Jürgens, G. The cytokinesis gene KEULE encodes a Sec1 protein that binds the syntaxin KNOLLE. *Journal of Cell Biology* (2001). 10.1083/jcb.152.3.531 PMC2195996 11157980

[60] Kennedy BW, Quinton M, van Arendonk JA (1992). Estimation of effects of single genes on quantitative traits. J. Anim. Sci..

[61] Nurul Haque Mollah, M., Sultana, N., Minami, M. & Eguchi, S. Robust extraction of local structures by the minimum β-divergence method. *Neural Networks* (2010). doi:10.1016/j.neunet.2009.11.011 10.1016/j.neunet.2009.11.011 19963342

[62] Mollah MMH, Jamal R, Mokhtar NM, Harun R, Mollah MNH (2015). A hybrid one-way ANOVA approach for the robust and efficient estimation of differential gene expression with multiple patterns. PLoS ONE.

[63] Zhang B, Kirov S, Snoddy J (2005). WebGestalt: an integrated system for exploring gene sets in various biological contexts. Nucleic Acids Res..

[64] Wang K, Abbott D (2008). A principal components regression approach to multilocus genetic association studies. Genet. Epidemiol..

[65] Li J, Zhong W, Li R, Wu R (2014). A fast algorithm for detecting gene-gene interactions in genome-wide association studies. Ann. Appl. Stat..

[66] Schwender H (2012). Imputing missing genotypes with weighted k nearest neighbors. J. Toxicol. Environ. Health Part A Curr. Issues.

[67] Sarkar, D. Package ‘lattice’: Trellis Graphics for R. (2017). http://lattice.r-forge.r-project.org/

[68] Gu Z, Eils R, Schlesner M (2016). Complex heatmaps reveal patterns and correlations in multidimensional genomic data. Bioinformatics.

[69] Liu L, Zhang Z, Mei Q, Chen M (2013). PSI: a comprehensive and integrative approach for accurate plant subcellular localization prediction. PLoS ONE.

[70] Wei, T. *et al.* R package ‘corrplot’: Visualization of a Correlation Matrix. (2017). https://github.com/taiyun/corrplot

