# Publisher Correction: Robustification of GWAS to explore effective SNPs addressing the challenges of hidden population stratification and polygenic effects

**DOI:** 10.1038/s41598-021-00399-z

**Published:** 2021-11-10

**Authors:** Zobaer Akond, Md. Asif Ahsan, Munirul Alam, Md. Nurul Haque Mollah

**Affiliations:** 1 Bioinformatics Lab, Department of Statistics, University of Rajshahi, Rajshahi, 6205 Bangladesh; 2 Institute of Environmental Science, University of Rajshahi, Rajshahi, 6205 Bangladesh; 3 Molecular Ecology and Metagenomic Laboratory, Infectious Diseases Division, International Centre for Diarrheal Disease Research (Icddr,b), Rajshahi, Bangladesh; 4 Agricultural Statistics and ICT Division, Bangladesh Agricultural Research Institute (BARI), Gazipur, 1701 Bangladesh

Correction to: *Scientific Reports* 10.1038/s41598-021-90774-7, published online 22 June 2021

The original version of this Article contained typographical errors in the Equations.

In Table 1, fourth column, the Equation “Polygenic effect variation” was incorrect:

$${\text{var}}\left( {\mathop \sum \limits_{{k - m_{1} + 1}}^{{{\text{m}}_{2} }} {\text{b}}_{{\text{k}}} {\text{Z}}_{{\text{k}}} } \right)$$

now reads:

$${\text{var}}\left( {\mathop \sum \limits_{{k = m_{1} + 1}}^{{{\text{m}}_{2} }} {\text{b}}_{{\text{k}}} {\text{Z}}_{{\text{k}}} } \right)$$

In the Materials and methods section, under the subheading “Robustification of LMM based GWAS by using the outlier modification rule (proposed)”,

“***b*** is the vector of random polygenic effects which follows *N(0,σ*_*g*_^*2*^*K)*, where *σ*_*g*_^*2*^ is the polygenic variance component, and *K* = *(k*_*ijt*_*)* is the *m* × *m* genomic relationship matrix.”

now reads:

“***b*** is the vector of random polygenic effects which follows *N(0,σ*_*g*_^*2*^*K)*, where *σ*_*g*_^*2*^ is the polygenic variance component, and *K* = *(k*_*jt*_*)* is the *m* × *m* genomic relationship matrix.”

And,

“When the maximum likelihood (ML) or restricted maximum likelihood (REML) variance component $\hat{\mathbf{V}}={\hat{\sigma }}_{g}^{2}\mathbf{K}+{\hat{\sigma }}_{\varepsilon }^{2}\mathbf{I}$ is estimated, the classical *F*-statistic for testing the null hypothesis *Ma* = *0* for an arbitrary full-rank *p* × *q* matrix *M*^13,60^.”

now reads:

“When the maximum likelihood (ML) or restricted maximum likelihood (REML) variance compontent $\hat{\mathbf{V}}={\hat{\sigma }}_{g}^{2}{\mathbf{Z}\mathbf{K}\mathbf{Z}}^{^{\prime}}+{\hat{\sigma }}_{\varepsilon }^{2}\mathbf{I}$ is estimated, the classical *F*-statistic for testing the null hypothesis *Ma* = *0* for an arbitrary full-rank *p* × *q* matrix *M*^13,60^.”

And,

“(ii) Divide the phenotypic data into *m* groups corresponding to the *m* genotypic labels of the selected most significant SNP. For example, let

$$ {\varvec{y}} = (y_{1} ,y_{2} ,...,y_{n} )^{\prime} = (y_{11} ,..,y_{{_{1} n_{1} }} ,...,\;y_{m1} ,..,y_{{mn_{m} }} )^{\prime} $$

be the partition of phenotypic observations corresponding to the selected SNP, where, *n* = *n*_*1*_ + *n*_*2*_ + *……..*+ *n*_*k*_.

(iii) Detect the outlying observations from the *l*^*th*^ (*l* = *1,2,…,k*) group using the *β*-weight function defined by

4 $${W}_{\beta }({y}_{li}|{\hat{\theta }}_{l})=exp \bigg \{-\frac{\beta }{2{\sigma }_{li}^{2}}({y}_{li}-{\hat{\mu }}_{l}{)}^{2}\bigg \}$$

where *i* =  1, 2, *…, n”*

now reads:

“(ii) Divide the phenotypic data into *m* groups corresponding to the *m* genotypic labels of the selected most significant SNP. For example, let

$$ {\varvec{y}} = (y_{1} ,y_{2} ,...,y_{n} )^{\prime} = (y_{11} ,..,y_{{_{1} n_{1} }} ,...,\;y_{m1} ,..,y_{{mn_{m} }} )^{\prime} $$

be the partition of phenotypic observations corresponding to the selected SNP, where, *n* = *n*_*1*_ + *n*_*2*_ + *……..* + *n*_*m*_.

(iii) Detect the outlying observations from the *l*^*th*^ (*l* = *1,2,….,k*) group using the *β*-weight function defined by

4 $${W}_{\beta }({y}_{li}|{\hat{\theta }}_{l})=exp \bigg \{-\frac{\beta }{2{\sigma }_{li}^{2}}({y}_{li}-{\hat{\mu }}_{l}{)}^{2} \bigg \}$$

where *i* = 1, 2,  *…, n*_*l*_*”*

Additionally, Equation 7 was incorrect:

“An outlying phenotypic observation *y*_*lk*_ in the *l*^*th*^ group is defined based on the *β*-weight function mentioned below:

7 $$W_{\beta } (x_{{jk}} |\hat{\theta }_{{j,\beta }} ) = \left\{ {\begin{array}{*{20}l} { > \tau _{j} ,\qquad {\text{if}}{\mkern 1mu} \;x_{{jk}} \;{\text{is}}\;{\mkern 1mu} {\text{not}}\;{\mkern 1mu} {\text{an}}\;{\mkern 1mu} {\text{outlier}}} \\ { \le \tau _{j} ,\qquad {\text{if}}\;x_{{jk}} \;{\mkern 1mu} {\text{is}}\;{\mkern 1mu} {\text{an}}\;{\mkern 1mu} {\text{outlier}}} \\ \end{array} } \right.$$

where the threshold value *τ*_*l*_ is the *p*^*th*^ quantile value of the empirical distribution of ${W}_{\beta }({x}_{li}|{\hat{\theta }}_{l,\beta })$.”

now reads:

“An outlying phenotypic observation *y*_*li*_ in the *l*^*th*^ group is defined based on the *β*-weight function mentioned below:

7 $$W_{\beta } (y_{{li}} |\hat{\theta }_{{j,\beta }} ) = \left\{ {\begin{array}{*{20}l} { > \tau _{j} ,\quad {\text{if}}{\mkern 1mu} \;y_{{li}} \;{\text{is}}\;{\mkern 1mu} {\text{not}}\;{\mkern 1mu} {\text{an}}\;{\mkern 1mu} {\text{outlier}}} \\ { \le \tau _{j} ,\quad {\text{if}}\;y_{{li}} \;{\mkern 1mu} {\text{is}}\;{\mkern 1mu} {\text{an}}\;{\mkern 1mu} {\text{outlier}}} \\ \end{array} } \right.$$

where the threshold value *τ*_*l*_ is the *p*^*th*^ quantile value of the empirical distribution of ${W}_{\beta }({y}_{li}|{\hat{\theta }}_{l,\beta }$).”

In Table 3, fourth column, the Equation “Polygenic effect variation” was incorrect:

$${\text{var}}\left( {\sum\limits_{{j = 1}}^{{{\text{m}}_{2} }} {{\text{b}}_{{\text{k}}} } {\text{x}}_{{\text{k}}} } \right)$$

now reads:

$${\text{var}}\left( {\sum\limits_{{k = m_{1} + 1}}^{{{\text{m}}_{2} }} {{\text{b}}_{{\text{k}}} } {\text{z}}_{{\text{k}}} } \right)$$

Under the subheading “Genotype simulation”,

“For this purpose, *m* = 2000 SNPs were generated for *n* = 1000 individuals, and these individuals were taken from *k* = 3 distinct population by considering different minor allele frequencies (MAFs). To do this, first, a set of *m*latent vectors {***v***_*1*_*, ****v***_*2*_*, …..****v***_*m*_} was generated from a multivariate normal distribution with mean zero and variance–covariance matrix Cov*(v*_*j*_,*v*_*k*_) = *ρ*^*|j-k|*^^64,65^. In our simulation, we considered *ρ* = 0.5 to avoid the linkage disequilibrium (LD) between the SNPs. Finally, two cutoff values *s*_*1*_ and *s*_*2*_ were used to convert the design matrix ***V*** = [***v***_*1*_*, ****v***_*2*_*, …, ****v***_*m*_ ] = [*v*_*ij*_] of latent vectors to the genotypic score matrix ${x}_{ij}(i={1,2},\ldots ,n,j={1,2},\ldots ,{m}_{1}){\text { and }}{z}_{ij}(i={m}_{1},1,\ldots ,n,j={1,2},\ldots ,{m}_{2})$ as follows:

$$x_{{ij}} ,z_{{ij}} = \left\{ {\begin{array}{*{20}l} { - 1,} & {v_{{ij}} < s_{1} } \\ {0,} & {s_{1} \le v_{{ij}} \le s_{2} } \\ {2,} & {v_{{ij}} > s_{2} } \\ \end{array} } \right.$$

where *s*_*1*_ and *s*_*2*_ determine the minor allele frequency.”

now reads:

“For this purpose, m* = 2000 SNPs were generated for *n* = 1000 individuals, and these individuals were taken from *k* = 3 distinct population by considering different minor allele frequencies (MAFs). To do this, first, a set of latent vectors {***v***_*1*_*, ****v***_*2*_*, …..****v***_m*_} was generated from a multivariate normal distribution with mean zero and variance–covariance matrix Cov*(v*_*j*_,*v*_*k*_) = *ρ*^*|j-k|*^^64,65^. In our simulation, we considered *ρ* = 0.5 to avoid the linkage disequilibrium (LD) between the SNPs. Finally, two cutoff values *s*_*1*_ and *s*_*2*_ were used to convert the design matrix ***V*** = [***v***_*1*_*, ****v***_*2*_***, …, v***_m*_ ] = [*v*_*ij*_] of latent vectors to the genotypic score matrix ${x}_{ij}(i={1,2},\ldots ,n,j={1,2},\ldots ,{m}_{1}){\text{ and }}{z}_{ij}(i=1,2,\ldots ,n;j={1,2},\ldots ,{m}_{2})$ as follows:

$$x_{{ij}} ,z_{{ij}} = \left\{ {\begin{array}{*{20}l} {0,} & {v_{{ij}} < s_{1} } \\ {1,} & {s_{1} \le v_{{ij}} \le s_{2} } \\ {2,} & {v_{{ij}} > s_{2} } \\ \end{array} } \right.$$

where *s*_*1*_ and *s*_*2*_ determine the minor allele frequency.”

Furthermore, in the section “Phenotype simulation,”

“In every situation, *m*_*1*_ = 4 SNPs were considered as causal variants and the remaining *m*_*2*_ = *m-m*_*1*_ = *1996* SNPs were allocated as polygenic variants (effects).”

now reads:

“In every situation, *m*_*1*_ = 4 SNPs were considered as causal variants and the remaining *m*_*2*_ = m* - m_*1*_ = *1996* SNPs were allocated as polygenic variants (effects).”

Lastly, in the section “Consequence of phenotypic outliers on the partition of total variations”,

$$h=\frac{{\text{var}}\left(\sum\limits_{k=1}^{{m}_{1}}{a}_{k}{x}_{k}\right)}{{\text{var}}\left({y}\right)}>\frac{{\text{var}}\left(\sum\limits_{k=1}^{{m}_{1}}{a}_{k}{x}_{k}\right)}{{\text{var}}\left({y}^{*}\right)}={h}^{2*}$$

now reads:

$${h}^{2}=\frac{{\text{var}}\left(\sum\limits_{k=1}^{{m}_{1}}{a}_{k}{x}_{k}\right)}{{\text{var}}\left({y}\right)}>\frac{{\text{var}}\left(\sum\limits_{k=1}^{{m}_{1}}{a}_{k}{x}_{k}\right)}{{\text{var}}\left({y}^{*}\right)}={h}^{2*}$$

The original Article has been corrected.

