# Supplementary material for: Robustification of GWAS to explore effective SNPs addressing the challenges of hidden population stratification and polygenic effects
Source: Sci Rep. 2021 Jun 22;11:13060. doi: 10.1038/s41598-021-90774-7 (PMC8219685; doi:10.1038/s41598-021-90774-7)
Supplement: Supplementary file 3 — Supplementary Information 3. [file 41598_2021_90774_MOESM3_ESM.doc]

Robustification of GWAS to Explore Effective SNPs Addressing the Challenges of Hidden Population Stratification and Polygenic Effects

Zobaer Akond^1, 2, 4, #^, Md. Asif Ahsan^1,#^, Munirul Alam^3^, Md. Nurul Haque Mollah^1^*

^1^Bioinformatics Lab, Department of Statistics, University of Rajshahi, Rajshahi-6205, Bangladesh

^2^Institute of Environmental Science, University of Rajshahi, Rajshahi-6205, Bangladesh

^3^Molecular Ecology and Metagenomic Laboratory, Infectious Diseases Division, International Centre for Diarrheal Disease Research, Bangladesh (icddr,b)

^4^Agricultural Statistics and ICT Division, Bangladesh Agricultural Research Institute (BARI), Gazipur-1701, Bangladesh

^#^These authors contributed equally to this work

*Corresponding author: Md. Nurul Haque Mollah (e-mail: mollah.stat.bio@ru.ac.bd)

**Supplementary Information**

**Table S1.** Identified 11 SNPs and the candidate genes and the genetic information of rice flowering time

| ID | P-value | Chr | Position | Locus | Distance | Start | End | Description |
| --- | --- | --- | --- | --- | --- | --- | --- | --- |
| id2005644 | 2.44E-05 | 2 | 12488337 | LOC_Os02g21070 | 4273 | 12492610 | 12493561 | PPR repeat domain containing protein, putative, expressed |
| id2005743 | 4.62E-05 | 2 | 13011782 | LOC_Os02g21880 | including | 13006951 | 13013160 | coiled-coil domain-containing protein, putative, expressed |
| id2005919 | 2.63E-07 | 2 | 13975952 | LOC_Os02g24134 | 1657 | 13977609 | 13987430 | Sec1 family transport protein, putative, expressed |
| ud2000772 | 1.82E-06 | 2 | 14370758 | LOC_Os02g24770 | including | 14368741 | 14371953 | retrotransposon protein, putative, Ty1-copia subclass, expressed |
| id2005983 | 4.88E-07 | 2 | 14376159 | LOC_Os02g24780 | including | 14374834 | 14379670 | retrotransposon protein, putative, unclassified, expressed |
| id2006587 | 8.89E-06 | 2 | 16434820 | LOC_Os02g27750 | including | 16432240 | 16438080 | transposon protein, putative, unclassified, expressed |
| wd6000761 | 3.44E-05 | 6 | 10471943 | LOC_Os06g18000 | 715 | 10469299 | 10471228 | protein kinase domain containing protein, expressed |
| ud7002027 | 8.82E-06 | 7 | 27420180 | LOC_Os07g45950 | 918 | 27421098 | 27423642 | expressed protein |
| id8000022 | 2.11E-05 | 8 | 51045 | LOC_Os08g01070 | 2995 | 54040 | 58330 | retrotransposon protein, putative, unclassified, expressed |
| id8004076 | 4.92E-05 | 8 | 15199041 | LOC_Os08g25040 | including | 15198870 | 15199151 | expressed protein |
| id8004083 | 3.14E-05 | 8 | 15206184 | LOC_Os08g25060 | including | 15203190 | 15211952 | BSD domain-containing protein, putative, expressed |

including indicates the protein-coding genes**.**

**Table S2. Identified SNPs located in the region of 100kb of the genes involved in flowering time and seed development.**

| ID | Gene | Pathway | Description |
| --- | --- | --- | --- |
| ud2000772 | LOC_Os02g24632 | Seed Development | Photosystem II 44 kDa reaction center protein, putative, expressed |
| id2005983 |
| id8000022 | LOC_Os08g01090 | Seed Development | B3 DNA binding domain containing protein, expressed |
| id8000022 | LOC_Os08g01054 | Flowering Time | retrotransposon protein, putative, unclassified, expressed |
| id8004076 | LOC_Os08g24946 | Flowering Time | EDM2, putative, expressed |


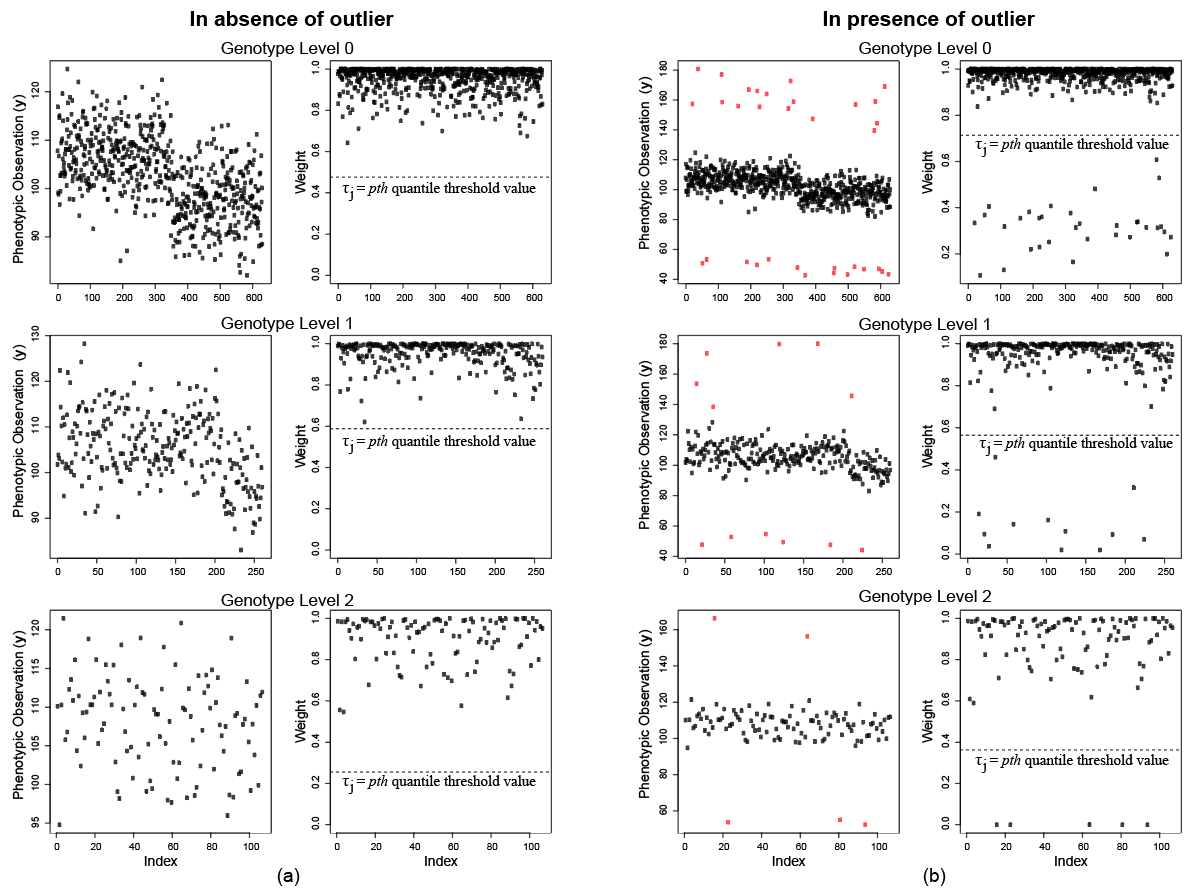


**Fig. S1. Outlier detection results by using the *β*-weight function for simulated SNP data.** (a) in absence of outlier (b) in presence of outlier. The scatter plots are for the phenotypic observations and the corresponding weights calculated with *β*-weight function at the genotype level 0, 1 and 2 of the identified most significant SNP using robust ANOVA.


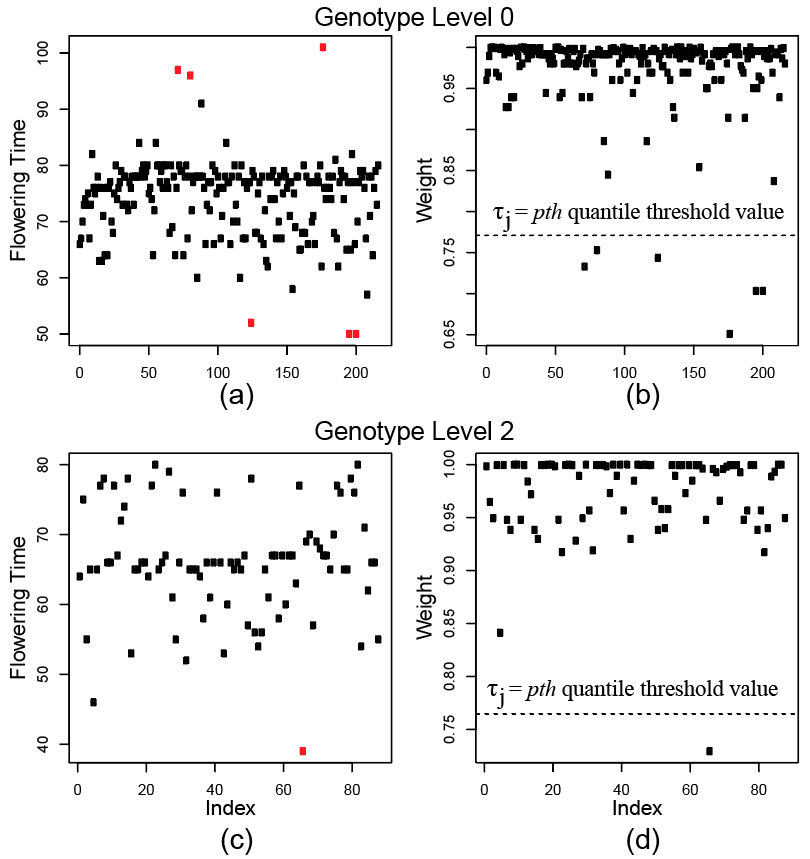


**Fig. S2**. **Outlier detection results by using the *β*-weight function for real rice flowering time SNP data.** The scatter plots are for the phenotypic observations (a and c) and the corresponding weights (b and d) calculated with *β*-weight function at the genotype level 0 and 2 of the identified most significant SNP using robust ANOVA.


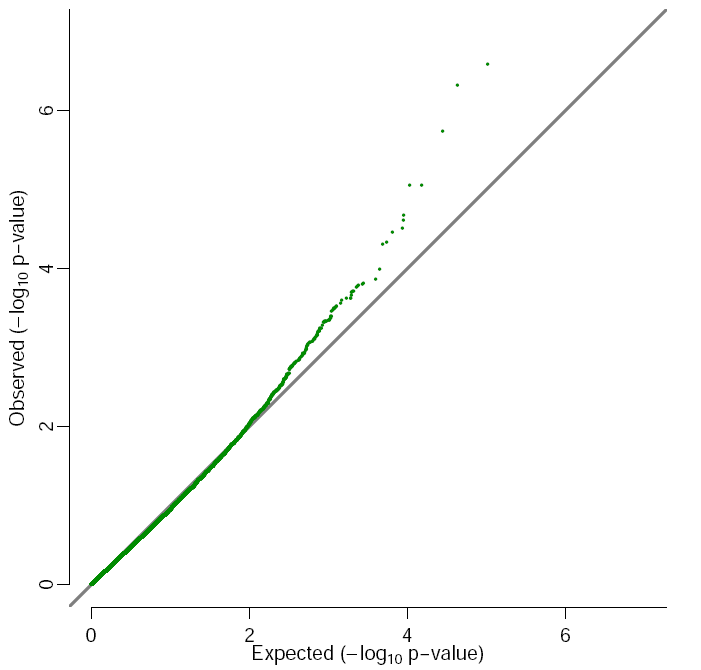


**Fig. S3. Quantile – Quantile plots (QQ-plots) for rGWAS for Flowering time.**


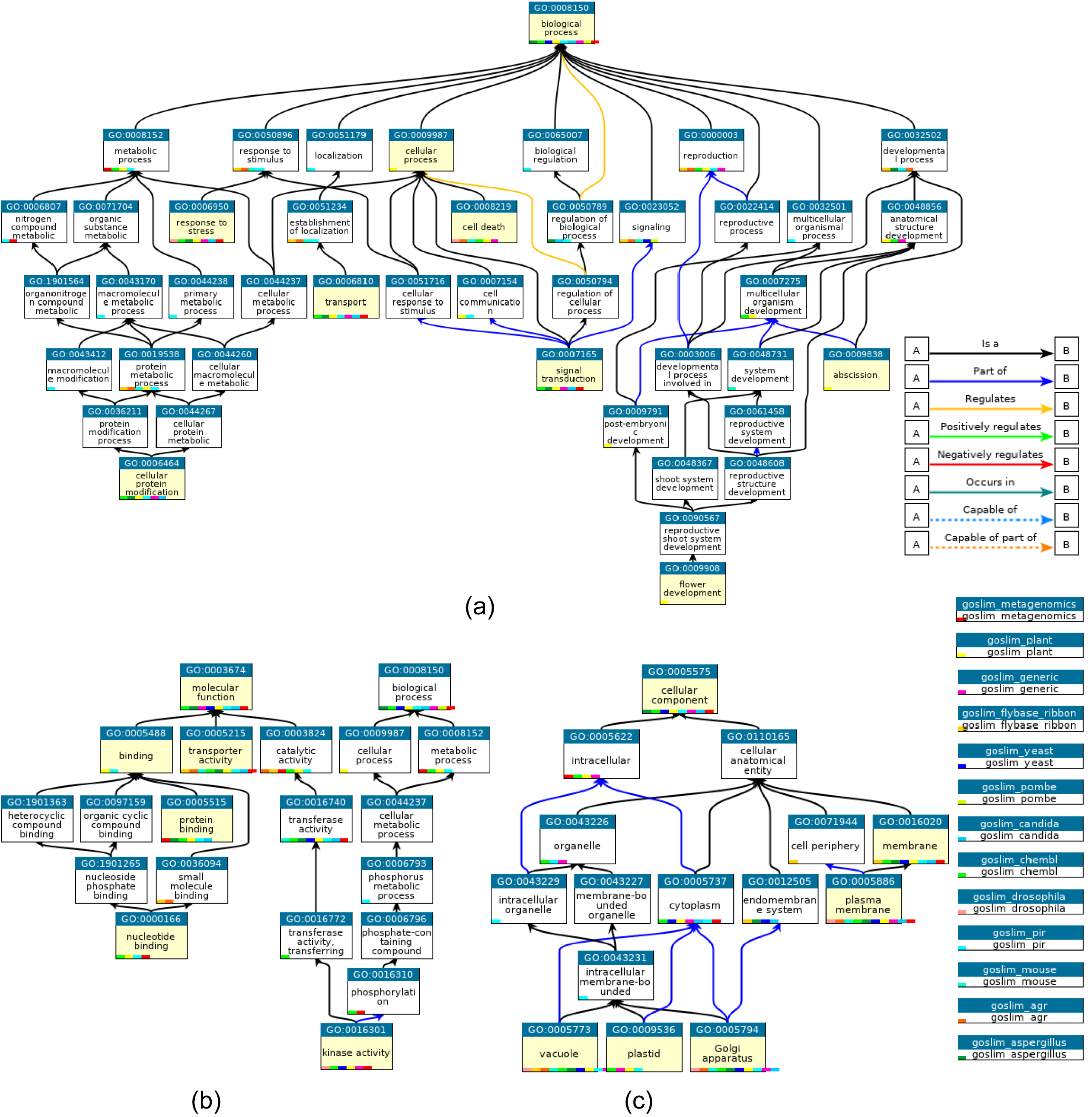


**Fig. S4.** GO (slim) annotations hierarchy tree of the predicted 11 SNP markers characterized in three categories (a) Biological Process (BP) (b) Molecular Function (MF) and (c) Cellular Component (CC). A web-based tool QuickGO was used to carry out the analysis of GO terms and annotations. Significantly associated functions (GO names) and GO terms are presented inside the light-yellow colored boxes of each category. Black arrow implies that ‘a type or sub-type of’ and blue arrow implies that ‘a part of’.
